# Agent-based evolving network modeling: a new simulation method for modeling low prevalence infectious diseases

**DOI:** 10.1007/s10729-021-09558-0

**Published:** 2021-05-15

**Authors:** Matthew Eden, Rebecca Castonguay, Buyannemekh Munkhbat, Hari Balasubramanian, Chaitra Gopalappa

**Affiliations:** Mechanical and Industrial Engineering, University of Massachusetts Amherst, Amherst, MA 01003 USA

**Keywords:** Agent-based simulation, Network modeling, Disease modeling, Scale-free networks

## Abstract

Agent-based network modeling (ABNM) simulates each person at the individual-level as agents of the simulation, and uses network generation algorithms to generate the network of contacts between individuals. ABNM are suitable for simulating individual-level dynamics of infectious diseases, especially for diseases such as HIV that spread through close contacts within intricate contact networks. However, as ABNM simulates a scaled-version of the full population, consisting of all infected and susceptible persons, they are computationally infeasible for studying certain questions in low prevalence diseases such as HIV. We present a new simulation technique, agent-based *evolving* network modeling (ABENM), which includes a new network generation algorithm, Evolving Contact Network Algorithm (ECNA), for generating scale-free networks. ABENM simulates only infected persons and their immediate contacts at the individual-level as agents of the simulation, and uses the ECNA for generating the contact structures between these individuals. All other susceptible persons are modeled using a compartmental modeling structure. Thus, ABENM has a hybrid agent-based and compartmental modeling structure. The ECNA uses concepts from graph theory for generating scale-free networks. Multiple social networks, including sexual partnership networks and needle sharing networks among injecting drug-users, are known to follow a scale-free network structure. Numerical results comparing ABENM with ABNM estimations for disease trajectories of hypothetical diseases transmitted on scale-free contact networks are promising for application to low prevalence diseases.

**Supplementary Information:**

The online version contains supplementary material available at 10.1007/s10729-021-09558-0.

**Highlights**

- A new agent-based evolving network modeling (ABENM) simulation technique.
- A new evolving contact network algorithm (ECNA) for generation of scale-free networks in ABENM.
- These new methods make it computationally feasible to model contact network structures for simulating epidemic spread of low prevalence infectious diseases; the low prevalence generates computational challenges when using current network simulation techniques.
- Low prevalence diseases could include current low-risk high disease burden epidemics such as HIV, TB, or Hepatitis B and C, or newly emerging diseases, such as the Ebola disease, or the Middle East Respiratory Syndrome, where rapid response during the early stages of an epidemic, when prevalence is low, is key for effective control.
- Network modeling allows for studying the dynamics of contact network structures for identifying effective intervention strategies, as a standalone method or when combining with new methods in other areas such as molecular cluster detection for evaluation of early detection and prevention strategies.

## Introduction

Mathematical simulation modeling can play a key role in predicting epidemic projections and informing response strategies for disease prevention through analyses of alternative intervention strategies. However, there is a lack of a suitable technique for simulating diseases that spread through defined contact structures but have low prevalence, including endemic diseases such as the human immunodeficiency virus (HIV) or tuberculosis (TB), or reemerging disease outbreaks such as Ebola disease or Middle East Respiratory Syndrome (MERS) where rapid response when prevalence is low is key. We present a new simulation technique, which we refer to as an agent-based evolving network modeling (ABENM) technique, and a new evolving contact network algorithm (ECNA) for generating scale-free networks in ABENM. This technique was specifically developed for analyses of diseases with low prevalence.

Current simulation techniques are insufficient for low prevalence diseases where the network structure of contacts significantly influences virus spread. Two commonly used types of simulation techniques in the disease modeling literature are compartmental modeling and agent-based network modeling (ABNM). [1] Compartmental modeling splits the population into groups (or compartments) that represent the different states of a disease, e.g., susceptible, infected, and removed, and uses a system of differential equations to simulate the rates of change for transitioning between these compartments. These models assume random mixing between people, which is suitable for diseases that spread easily through air droplets such as the seasonal flu but not suitable for diseases that spread through close contact networks such as HIV, Ebola disease, MERS, and TB. [2] ABNM simulates each entity as an individual agent or node in a network and the connections between nodes as links of a network. ABNM is thus suitable for simulating infected and susceptible persons at the individual-level and the interactions leading to disease transmissions through a network of contacts, [3] representative of say sexual and needle-sharing partnerships that are the most common modes of HIV transmission, or close family members for TB, Ebola disease, and MERS. However, ABNM is computationally expensive. Therefore, while ABNM provides the necessary mathematical structure, it is computationally infeasible for diseases with low prevalence. Taking HIV as an example, about 421 per 100,000 persons are infected with HIV nationally in the United States (U.S.), [4] and the size of the underlying contact networks are expected to be 2 to 49 persons per cluster as per molecular analyses of nucleotide sequence data from persons with recent diagnoses (i.e., those diagnosed over the past 3 year period, who constitute about 10% of all PWH). [5] As ABNM are scaled versions of the population, simulating a population of 100,000 persons representative of the U.S. population will have 421 HIV-infected persons, and 42 persons with recent diagnosis, which are insufficient to generate the expected cluster sizes or model heterogeneity by key features, such as risk-group, age, and race. Computation times in ABNM are in the order of *O*(*N*^2^), where *N* is the population size in the simulation, as such, increasing the value of *N* is also not a suitable solution. These issues make ABNM insufficient for modeling low prevalence diseases such as HIV.

The key aspect of the newly developed ABENM is to only simulate infected individuals and their network of infected and susceptible contacts at the individual-level, and all other susceptible persons at the population-level as a compartmental model. As susceptible persons become infected, their contacts will be introduced to the network, thus the size of the network grows as new persons become infected. The ECNA is a network generation algorithm that determines the degree (the number of contacts) of each newly added person such that the resulting network would match overall network statistics. This method enables modeling low prevalence diseases on network structures and gives a good trade-off between the current two extreme simulation techniques.

Among the computational modeling literature using scale-free networks, several focused on non-network features such as various types of interventions. [6–12] Two studies further focused on different features related to sexual behavior, highlighting the significance of modeling network structures. [7, 8] One study, on computer viruses, focused on understanding the influence of structural properties of scale-free networks on epidemic spread. [13] Two studies developed open source generalized modeling frameworks, FAVITES for studying physical contact networks and molecular phylogenetic trees and sequences, and SimpactCyan an open-source agent-based simulation tool for HIV with R and Python interfaces. [14, 15] Two studies also focused on development of methods for calibration of the models rather than network features. [6, 15]

All the above models simulate the full population, i.e., infected and susceptible persons, except for one study, that keeps track of only infected persons as agents, [11] but they did not explicitly simulate contacts as agents only as features of the infected persons. The motivation of their methodology is similar to our work, modeling low prevalence diseases, however, they do not track or model the contact network and thus it limits its use for studying the transmission dynamics on contact network structures. Our work addresses this gap.

Two of the other studies discussed above highlight the limitations caused by constraints in memory and computational times generated by the use of network modeling. One study noted that the computational times are generally in the order of *O*(*N*^2^) per time-step and in the worst case go up to *O*(*N*^3^). [6] They simulate a population generally starting at 100,000 agents and reaching utmost 750,000 agents after 12 years, with each run taking about 3 to 6 h on a single processor. However, this model was applied to South Africa where the HIV prevalence is much higher, about 15 to 35% in the last year of the simulation. The SimpactCyan model was demonstrated on population sizes of 5000 to 20,000 and the study reported a runtime of 25–45 min on a single core. [15]

All other models did not discuss computational complexity but used small population sizes or assumed static networks, and are applied to or demonstrated on high prevalence populations. Moshiri et.al. (2019), used preferential attachment to generate a scale-free contact network for simulating HIV through a static sexual contact network [14]. They apply the model for simulating HIV in San Diego and Uganda, using network sizes of 10,000 or 100,000, and starting with an initial HIV prevalence of 15%. Pastor-Satorras and Vespignani (2000) used large network sizes of upto 8,500,000 to study the spread of computer viruses on static-networks, which do not face similar computational complexities as dynamic networks [13]. Kretzschmar and Morris (1997) use population size of 2000 [8]. Vieira et al. (2010) use small world networks for simulating HIV in a small Brazilian population of about 3400 [10]. Johnson et.al. (2018), start with an initial population size of 20,000 with a prevalence of 1–3% and simulate to a final prevalence of 30% [9]. Luo et al. (2018), initialize the model with 10,000 persons and simulate MSM in Atlanta, a population with high HIV prevalence [12]. Reniers et.al. (2015), simulated a small population of 1250 persons starting with an initial prevalence of 5% to model sexual behavior and partnership networks, in the context of HIV in Africa [7].

Methods in the social network literature for network generation, including network evolution models [16–19], nodal attribute models [20], preferential attachment [19], and exponential random graph models [21, 22], mostly focus on addition of heterogeneous features and network statistics into network generation. These algorithms generate the full population network and thus are suitable for generating networks for ABNM simulations only.

Therefore, the significance of the newly proposed ABENM and ECNA is the focus on modeling network structures for diseases with low prevalence that are spread on non-random networks. This is a category that is not explicitly studied in prior computational epidemiology or social network modeling literature but representative of a significant portion of high burden diseases and is challenging to model with current techniques. This is a concept paper for presenting the ABENM simulation technique with ECNA network generation algorithm specifically for scale-free contact networks where node degree distribution follows a power-law [13, 23]. Networks of this type include needle sharing contact networks among people who inject drugs and sexual contact networks [24–27].

## Methods

### Problem description

**Fig. 1:**
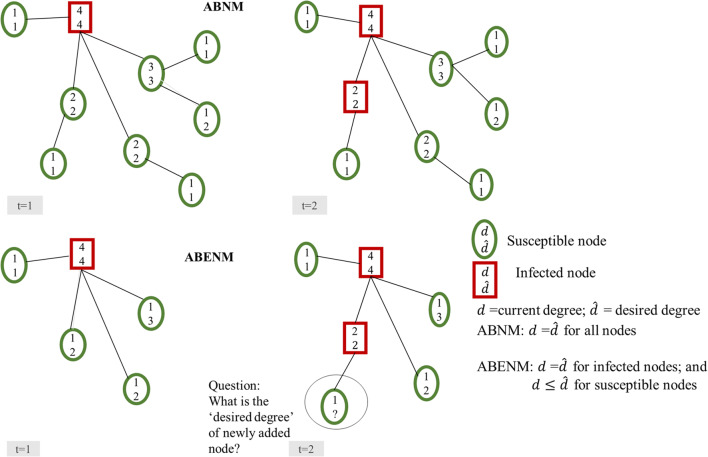
Overview of structural differences between the agent-based network modeling (ABNM) and our proposed agent-based evolving network modeling (ABENM) techniques, using a small network of size 9, at two time steps, t = 1 and t = 2, of the simulation. In ABNM, the network is first generated such that the degree of all nodes are known before the start of the simulation. In ABENM, only infected persons and immediate contacts are tracked. At every time-step, for every newly infected node, the desired degree of its newly added susceptible contacts need to be determined, which is the focus of the proposed evolving contact network algorithm (ECNA); current degree = number of current contacts (edges) of the node; desired degree = actual degree o f the node

In ABNM, the network of contacts is first generated using a network generation algorithm such as preferential attachment for scale-free networks, and transmission of infections are generated through simulation of individual-level interactions between contacts. In the empirical example in Fig. 1 for a hypothetical population of 9 people, all 9 persons (nodes) and their contact structure are initially generated and the spread of infection is simulated over time. The equivalent for the newly proposed ABENM, as shown in Fig. 1, models only infected nodes and their immediate contacts. Therefore, at every time-step, when a new person becomes infected, the problem is to determine the contacts of the newly infected node, specifically, ‘what is the degree (number of contacts) of the neighbors of the newly infected node?’ Errors in this estimation can lead to inaccurate epidemic trajectories, and inaccurate network structures.

We hypothesize that 1) current network generation algorithms, such as the preferential attachment which is the most commonly used algorithm for scale-free networks, cannot be used for ABENM; 2) the degree of node neighbors cannot be directly drawn from the overall network degree distributions due to known degree-correlations between nodes, i.e., the degree of a node is not independent of its neighbors’ degree, 3) current estimations for degree correlations are insufficient because of the overlying effects of the stochastic process defining the epidemic trajectory thus requiring new stochastic estimations, and 4) that the estimations are different for static and dynamic contact networks. The technical terms are discussed in more detail in the following sub-sections.

The rest of this section is structured as follows. We first discuss the mathematical formulations of the two commonly used simulation modeling techniques for prediction of disease trajectories during epidemics: a) deterministic compartmental modeling in Section 2.1; and b) agent-based network modeling (ABNM), including an overview of agent-based modeling in 2.2 and network generation algorithms in 2.3. We then present the newly developed simulation technique, ABENM, its overall framework in 2.4 and an algorithm for generating an evolving network, ECNA, in 2.5. We discuss our approach to validation of the ABENM with ECNA in 2.6.

### Overview of current compartmental simulation modeling technique

Compartmental modeling is a simulation modeling technique that is extensively used for epidemic predictions [1]. Using the simplest epidemic structure, Susceptible-Infected-Removed (SIR), a compartmental model for simulating the epidemic trajectory, specifically, estimating *s*_*t*_, *i*_*t*_, *r*_*t*_, the proportion of people in states S, I, and R, respectively can be written as

1 $$ {s}_t={s}_{t-1}-{s}_{t-1}p{c}_{t-1}{i}_{t-1}-{s}_{t-1}{\mu}_S $$

2 $$ {i}_t={i}_{t-1}+{s}_{t-1}p{c}_{t-1}{i}_{t-1}-{i}_{t-1}{\mu}_I $$

3 $$ {r}_t={r}_{t-1}+{s}_{t-1}{\mu}_S+{i}_{t-1}{\mu}_I $$

where,

*p* : probability of transmission per susceptible-infected contact
*c*_*t*_ : average number of contacts per person at time *t*
*c*_*t*_*i*_*t*_: average number of infected contacts per person at time *t*
*μ*_*S*_ : rate of transitioning from state *S* to *R* (in the case of HIV it represents natural mortality rate)
*μ*_*I*_ : rate of transitioning from state *I* to *R* (in the case of HIV it represents mortality rate from the disease)

A more generalized derivation of the compartmental model is presented in Appendix 1a, which can be extended to other types of epidemic profiles. Without loss of generality, and as typically done in compartmental model, instead of using *s*_*t*_, *i*_*t*_, *r*_*t*_ as the proportion of people in states S, I, and R, respectively, where *s*_*t*_ + *i*_*t*_ + *r*_*t*_ = 1 , we can also write these equations using *S*_*t*_, *I*_*t*_, *R*_*t*_ as the number of people in states S, I, and R, respectively, with *S*_*t*_ + *I*_*t*_ + *R*_*t*_ = *N*, where *N* is the size of total population.

Some terms from the above equations can then be defined epidemiologically as follows:

*Ns*_*t*__−1_*pc*_*t*_ _−1_ *i*_*t*−1_: Number of new infections at time *t*
*Ns*_*t* − 1_*μ*_*S*_: Number of new deaths among susceptible persons at time *t*
*Ni*_*t* − 1_*μ*_*I*_: Number of new deaths among infected persons at time *t*

As evident from the above equations and the generalized formulation in Appendix 1a, compartmental models do not simulate persons and their contacts individually, resulting in the assumption of random-mixing between individuals.

### Overview of current agent-based network modeling (ABNM) simulation technique

In ABNM, every person in the population is simulated at the individual-level, and the contacts between them are represented as links using network modeling. Each person is assigned a disease state, which is updated every time-step using individual-level features that influence disease state. For the simplest SIR epidemic structure, a person would be in one of S (susceptible), I (infected), or R(removed) state. Every time-step, it is determined if a susceptible person *j* would become newly infected (change from S to I) using the inverse of a Bernoulli distribution ${F}^{-1}\left(1-{\left(1-p\right)}^{{\overline{c}}_j}\right),$ where, *p*= probability of transmission per susceptible-infected contact (same as in compartmental model), ${\overline{c}}_j=$number of infected contacts of susceptible person *j* (determined individually for each person using the contact network), and $$ {F}^{-1}(a)=\left\{\begin{array}{c}1, if\ Uniform\left[0,1\right]<a\ \\ {}0,o/w\end{array}\right) $$. Similarly, it is determined if a person would change from S to R or I to R based on probabilities of natural deaths or disease-related deaths, respectively. The overall epidemic is represented as the proportion of people in each state.

### Overview of current network generation algorithms for ABNM

In ABNM, the network of contacts is generated prior to the epidemic simulation using network generation algorithms. The key purpose of network generation algorithms is to generate networks whose properties, e.g., degree distribution, match the properties observed in real world networks. In the context of a network, we define a *node* as an individual person, and an *edge* between two nodes as a contact between the two persons, say, representing sexual or needle-sharing partners in the case of HIV. The *degree* (*k*) of a node is the number of edges or links the node has with other nodes. The *degree distribution* of the network is the probability distribution of node degree. The type of distribution is dependent on the network, here we focus on scale-free networks where degree distribution follows a power law, [23] and thus the probability that a node has degree *k* (represented as *P*(*k*)) can be written as

$$ P(k)=C\ {k}^{-\lambda } $$

where, the decay-coefficient $\lambda =-\frac{\Delta \mathrm{log}\left({n}_k\right)}{\Delta \mathrm{log}(k)},{n}_k=$ number of nodes with degree *k*, Δ is the gradient, and *C* is a normalizing constant; for a given network, *λ* is a constant, making it a ‘scale-free’ network. The area of network science presents multiple algorithms for generating different types of networks. An algorithm commonly used for generating scale-free networks is the Barabási-Albert preferential attachment algorithm. [19, 28]

### Proposed agent-based evolving network modeling (ABENM) simulation technique

The newly developed agent-based evolving network modeling (ABENM) simulation technique combines the above theories of compartmental modeling and ABNM techniques. Specifically, we model infected persons and their immediate contacts as a network defined by the following parameters.

$$ {A}_t $$
a static adjacency matrix with dynamically changing size *Q*_*t*_ × *Q*_*t*_,where *Q*_*t*_ is the number of people modeled at the individual-level (i.e., only infected persons and their immediate contacts) at time *t*, and represents long-term contacts, e.g., for HIV, $\sum \limits_j{A}_{t, ij}$ would represent the number of lifetime partnerships of person *i*,
*V*_*t*_: a dynamic adjacency matrix of dynamically changing size *Q*_*t*_ × *Q*_*t*_, i.e., *V*_*t*, *ij*_ = 1 if contacts between *i* and *j* are active at time *t*, and 0 otherwise, and *V*_*t*, *ij*_≤ ${A}_{t, ij}$, e.g., ${A}_{t, ij}=1$ if *i* and *j* are needle sharing contacts, but *V*_*t*, *ij*_ = 0 is there was no needle sharing at time step *t*,
$$ {h}_{\mathrm{t}} $$
a row vector of size *Q*_*t*_ with each element *j* taking a binary value, 1 if person *j* is infected and 0 otherwise,
$$ {m}_t $$
a row vector of size *Q*_*t*_ with each element *j* taking a binary value, of 1 if person *j* is deceased and 0 otherwise,
$$ {u}_{\mathrm{t}} $$
a unit row vector of size *Q*_*t*_, and
$$ {c}_t $$
a row vector of size *N* with the value of element *j* equal to the number of active infected contacts of person *j* if *j* is susceptible and alive and zero otherwise, calculated as ${c}_t=\left({u}_{\mathrm{t}}-{h}_{\mathrm{t}}\right){}^{\circ}\left({u}_{\mathrm{t}}-{m}_t\right){}^{\circ}{\left({V}_t{h}_{\mathrm{t}}^{\mathrm{T}}\right)}^T$, where *T* denotes transpose and ° is elementary-wise multiplication.

One, at any time-step *t*, a susceptible node (say *j*) in the network becomes infected if ${F}^{-1}\left(1-{\left(1-p\right)}^{c_{t-1,j}}\right)=1$. Upon determining nodes that become newly infected, their contact structure is generated by incrementing *Q*_*t*_ by the number of new infections, i.e., ${Q}_t={Q}_{t-1}+\left(\sum \limits_{j=1:{Q}_{t-1}}{F}^{-1}\left(1-{\left(1-p\right)}^{c_{t-1,j}}\right)\right)$and updating all elements of the network described above, including ${A}_t$, *V*_*t*_, and ${c}_t$ by applying the ECNA (described in 2.5) to determine ‘who’ the contacts are of the newly infected nodes. Susceptible persons who are not contacts of infected persons would be tracked as in compartmental model, (4) below, and the overall epidemic projections can be written as follows, by replacing *s*_*t* − 1_*pc*_*t* − 1_*i*_*t* − 1_ in (1) and (2) with $\frac{\sum \limits_{j=1:{Q}_{t-1}}{F}^{-1}\left(1-{\left(1-p\right)}^{c_{t-1,j}}\right)}{\mathrm{N}}$in (4) and (5) and *i*_*t* − 1_ in (2) and (3) with $\frac{h_{t-1}{u}_{\mathrm{t}}^T}{N}$ in (5) and (6). More detailed derivations of ABENM from ABNM are in Appendix 1.

4 $$ {s}_t={s}_{t-1}-\frac{\sum \limits_{j=1:{Q}_{t-1}}{F}^{-1}\left(1-{\left(1-p\right)}^{c_{t-1,j}}\right)}{\mathrm{N}}-{s}_{t-1}{\mu}_S $$

5 $$ {i}_t=\frac{h_{t-1}{u}_{\mathrm{t}}^T+\sum \limits_{j=1:{Q}_{t-1}}{F}^{-1}\left(1-{\left(1-p\right)}^{c_{t-1,j}}\right)-{\mu}_I{h}_{t-1}{u}_{\mathrm{t}}^T}{N} $$

6 $$ {r}_t={r}_{t-1}+{s}_{t-1}{\mu}_S+\frac{\mu_I{h}_{t-1}{u}_{\mathrm{t}}^T}{N} $$

Without loss of generality, we can extend this ABENM structure developed for SIR on a closed population to other epidemic structures, incorporate heterogeneity, and model an open population. As demonstration, we present ABENM for one additional epidemic structure, one SIR structure with heterogeneity, and an SIR model with births and deaths, in Appendix 2a, 2b and 2c, respectively. A step-by-step algorithm for simulating the epidemic trajectories is outlined in Table 1. Step 4 of the ABENM simulation in Table 1 uses a new network generation method which we describe next.

**Table 1:** Overview of the ABENM for simulating epidemic trajectories for a SIR model: predicting the proportions susceptible, infected, and recovered (*s*_*t*_, *i*_*t*_, *r*_*t*_) as a function of time *t*

Step 1	Initial setup for *t = 0*:
1a:	Set the initial values for proportions susceptible, infected, and recovered, i.e., values for *s*_*t* = 0_*, i*_*t* = 0_, and *r*_*t* = 0_, respectively. For the example for HIV, we could set *i*_0_ as prevalence in base year of analyses, *r*_0_ as zero, and *s*_0_ as 1 − *i*_0_.
1b:	Based on the computational and sample size requirements, determine the total population size (*N*) and convert the proportions *s*_*t*_*, i*_*t*_, and *r*_*t*_ to numbers of people.
1c:	Determine *Q*_*t* = 0_, the initial number of people to model at the individual-level, and ${A}_{\mathrm{t}=0}$, an adjacency matrix representing their contact structure. We do not discuss the initial network generation method as this is a separate problem, which also arises even in ABNM. One method is to start with 1 infected person and do a dry run until the required i_t_ proportion of people are infected. For reference, in the Progression and Transmission of HIV (PATH 2.0) agent-based model developed in our previous work, they were estimated by calibration to multiple distributions of individual-level characteristics from surveillance.
1d:	Generate degree distribution vectors, *v* and $\hat{v}$, as follows • $\hat{v}$= a vector that keeps track of the degree distribution of the partial network defined by ${A}_{\mathrm{t}}$, and *v* = a vector that keeps track of the degrees of the *N* − *Q*_*t*_ persons not in ${A}_{\mathrm{t}}$ • ${\hat{v}}_k$= element k of the vector $\hat{v}$ = number of agents among the *Q*_*t*_ agents in the simulation who have a degree k • *v*_*k*_ = element k of the vector *v =*$P(k)\ N-{\hat{v}}_k$, where *P*(*k*) is the probability that a node in the full network has degree k, for scale-free networks it follows a power law distribution • ∣*v*∣ *=*$\mid \hat{v}\mid$ = size of the vectors = the maximum degree in the network, Therefore, *v*_*k*_ keeps track of the number of nodes of degree k who are not yet an agent in the simulation, i.e., persons who are susceptible and not contacts of infected persons
Step 2:	Determine transmissions from infected persons to immediate contacts at the individual-level using a Bernoulli transmission model
Step 3:	Calculate *s*_*t*_, *i*_*t*_, *r*_*t*_,using the model in (4), (5), and (6)
Step 4:	Evolve the network, specifically, determine the degree for the contacts of the newly infected persons. We develop a new algorithm that we refer to as the ‘evolving contact network algorithm’(ECNA), discussed in Table 2 below
Step 5:	Increment t. Stop if reached end of simulation time step, if not, go to Step 2

### Evolving contact network algorithm, a new network generation method for ABENM (step 4 of ABENM in Table 1)

The main objective of network generation methods, generally in the area of network science, is to determine who should be linked to who based on their degrees, such that the degree distribution of the resulting network matches that of the network it is replicating. The main objective of a network generation method for the ABENM is to determine the degree of the contacts of the newly infected persons, thus it is the main method that ‘evolves’ the network. We will refer to this network generation method as the ‘evolving contact network algorithm (ECNA)’. Before presenting the ECNA, we first discuss current network generation algorithms and its inapplicability to ABENM, and some relevant network properties that inform the development of the ECNA.

Current methods, such as the commonly used preferential attachment (PA) algorithm [28] for generation of scale-free networks cannot be used for the ECNA network generation. The PA algorithm starts with a small, say *m*_0_, number of initial connected nodes. Then, new nodes are added to the network one at a time by connecting them to *m* ≤ *m*_0_ existing nodes, *m* is the minimum degree of the network. This is continued until all nodes of the population are added to the network. The probability, *p*(*k*_*i*_), that a new node will connect to node *i* depends on the degree of the node *k*_*i*_, i.e., $p\left({k}_i\right)=\frac{k_i}{\sum \limits_{j=1:N}{k}_j}$, where *N* is the number of nodes in the network at that point. In this method, the degree of each node is evolving as new nodes are added and ‘preference’ of attachment goes to nodes that have higher contacts.

On the other hand, for the ABENM simulation, the objective of a network generation algorithm is to determine the expected degree of the immediate node neighbors of a newly infected person. The ABENM starts with an infected node and its immediate node neighbors (both infected and susceptible contacts) in the network. If there is a transmission from an infected node to their susceptible node neighbor, the algorithm should determine the expected degree of the immediate node neighbors’ of the newly infected node.

For non-random graphs such as scale-free networks, the expected degree of node neighbors cannot be directly drawn from the probability mass function of the network degree distribution. [29] This is because of degree correlations between node neighbors, i.e., the degree of two nodes who are connected (node neighbors) are not independent of each other but are correlated. Degree-correlation is usually measured as the probability that a randomly chosen neighbor of a node of degree *k* will have degree *l*, denoted as *Pr*(*L* = *l*| *K* = *k*). The conditional degree distribution *Pr*(*L* = *l*| *k*), is the probability distribution of *L* given specific *k*, i.e., the distribution of the degree of all nodes that are connected to a node of degree *k*. This distribution, in several real-world networks, is found to not follow the network degree distribution but be dependent on *k* [29] i.e., *Pr*(*L* = *l*| *K* = *k*) ≠ Pr(*L* = *l*). Degree-correlations are a well-studied area in graph theory from the perspective of understanding network properties such as assortative or dis-assortative mixing, or studying shortest paths or all paths between any two nodes. Fotouhi and Rabbat, 2013, [29] presented an analytical model for this conditional distribution generally for scale-free networks, and is summarized in Appendix 1c.

#### Conditional probability distributions derived from general scale-free networks (non-contagion) are not suitable for the ECNA

We hypothesize that conditional distributions derived for general scale-free networks cannot be used for determining the degree of the neighbors of the newly infected nodes in the ECNA network generation, which we present as Remarks 2 and 3 in Appendix 3, and provide an intuitive discussion here. The analytical expression for the conditional probability distribution derived on a general scale-free network would be representative of the distribution of degree of node neighbors of a randomly chosen set of nodes in the network. Empirically, the data for this can be generated by starting with one node, collecting their degree and the degree of each of their neighbors, and repeating this for all nodes. Therefore, if we consider nodes A and B in an undirected graph, the degree of A given degree of B and the vice-versa, i.e., the degree of B given the degree of A, are both incorporated into the estimation of the probability mass function. However, in the case of epidemics, the chance of A infecting B versus B infecting A would not be equal but vary as a function of the degree of A and B and the prevalence (proportion of population infected) at that time-point, thus creating directionality in flow (epidemic path) and making the chance of infection non-stationary as the prevalence changes over time, and should be thus considered in estimation of conditional distributions for ECNA. The above reasoning also drives the concept that highly connected nodes get infected sooner than nodes with fewer connections, which has been studied through the use of shortest paths and centrality measures. However, though the reasoning is similar, the expression for the conditional distribution of the degree of node neighbors considering the directionality and non-stationarity generated because of the stochasticity of an epidemic has not been evaluated. Therefore, we conducted numerical experiments to present our hypothesis, which we present in the next section. We refer to general scale-free networks as static non-contagion networks, and networks in the context of studying epidemic paths as contagion networks.

#### Numerical testing of degree correlations in networks along epidemic paths

We compared theoretical estimates of *Pr*{*L* = *l*| *k*} with numerical estimations, with theoretical estimates calculated using the expression derived in [29] (presented in Appendix 1c). Numerical estimations were conducted through ABNM simulations, of the type discussed in Section 2.2. and 2.3, as follows. We used the *barabasi.game* function in R software to generate scale-free networks. By varying the function input for minimum degree (*m*) between 1 to 5, and varying population sizes (*N*) of 1000 and 10,000 (*N* and *m* are the inputs to the *barabasi.game* function to generate networks of different *λ*) we generated scale-free networks of *λ* between 2 to 3, which is the typical range used for scale-free networks [28]. We generated 100 simulations for each value of *m*. We initiated the simulation with a small number of infected nodes, and at every time step simulated transmission from an infected node to its susceptible neighbors with some probability, thus generating epidemic paths and gathering the degree of every pair of node neighbors on these paths. Specifically, for every newly infected person with degree *K* = *k*, we updated the counter *z*_*l* ∣ *k*_, which is the number of neighbors with degree *l*. We estimated the distributions $\mathit{\Pr}\left\{L=l|k\right\}=\frac{z_{l\mid k}}{\sum \limits_{l=1:M}{z}_{l\mid k}}$ for every *k*, *l* combination, where *M* is the maximum network degree, under the following 3 scenarios:

1. Non-contagion networks: This scenario is formulated to estimate degree correlations in non-contagion networks, by moving through all paths. This is done by setting the probability of transmission as 1, such that the probability of transmission to any node connected to at least one infected neighbor would be 1. This setting counts all neighbors of the newly infected node in updating the counter *z*_*l* ∣ *k*_.
2. Static contagion networks: This scenario is formulated to estimate degree correlations along epidemic paths when the contact network is static. Epidemic paths are generated by setting 0 < *p* < 1 and calculating the probability of transmission for a node *j* using a Bernoulli model as $1-{\left(1-p\right)}^{\sum \limits_{q=1:{d}_j}{h}_q},$where *d*_*j*_= the number of contacts of node *j* and *h*_*q*_ = 1 if the *q*^*th*^ contact of node *j* is infected. This setting replicates a static contact network as all contacts of an infected node are active and thus transmission is directly proportional to a node’s degree (corresponds to model in Remark 2 in Appendix 3). This setting counts only susceptible neighbors of the newly infected node in updating the counter *z*_*l* ∣ *k*_.
3. Dynamic contagion networks: This scenario is formulated to estimate degree correlations along epidemic paths when the contact network is dynamic. Epidemic paths are generated by setting 0 < *p* < 1 and calculating the probability of transmission for a node *j* using a Bernoulli model as $1-{\left(1-p\right)}^{\sum \limits_{q=1:{d}_j}{h}_qf\left({d}_q\right)},$ where *d*_*j*_= the number of contacts of node *j*, *h*_*q*_ = 1 if the *q*^*th*^ contact of node *j* is infected and 0 otherwise, *d*_*q*_ is the degree of the *q*^*th*^ contact, and $$ f\left({d}_q\right)=\left\{\begin{array}{c}1\  if\ {F}^{-1}\left(U\left[0,1\right]\right)<\frac{1}{d_q}\\ {}0\  otherwise\end{array}\right. $$, and is 0 otherwise. This assumes that on average only one contact per person is active at any time and the chance of that active contact being node *j* is $\frac{1}{d_q}$. This setting thus replicates a dynamic contact network, where contacts are not active at all times. Note that in this network, the chance of transmission is directly proportional to its degree and inversely proportional to its neighbors’ degrees (see Remark 3 in Appendix 3). An example of a dynamic contact network would be needle sharing contacts among injecting drug users, where a person might share needles only on some days and with different people in their contact network. This setting counts only susceptible neighbors of the newly infected node in updating the counter *z*_*l* ∣ *k*_.

**Fig. 2:**
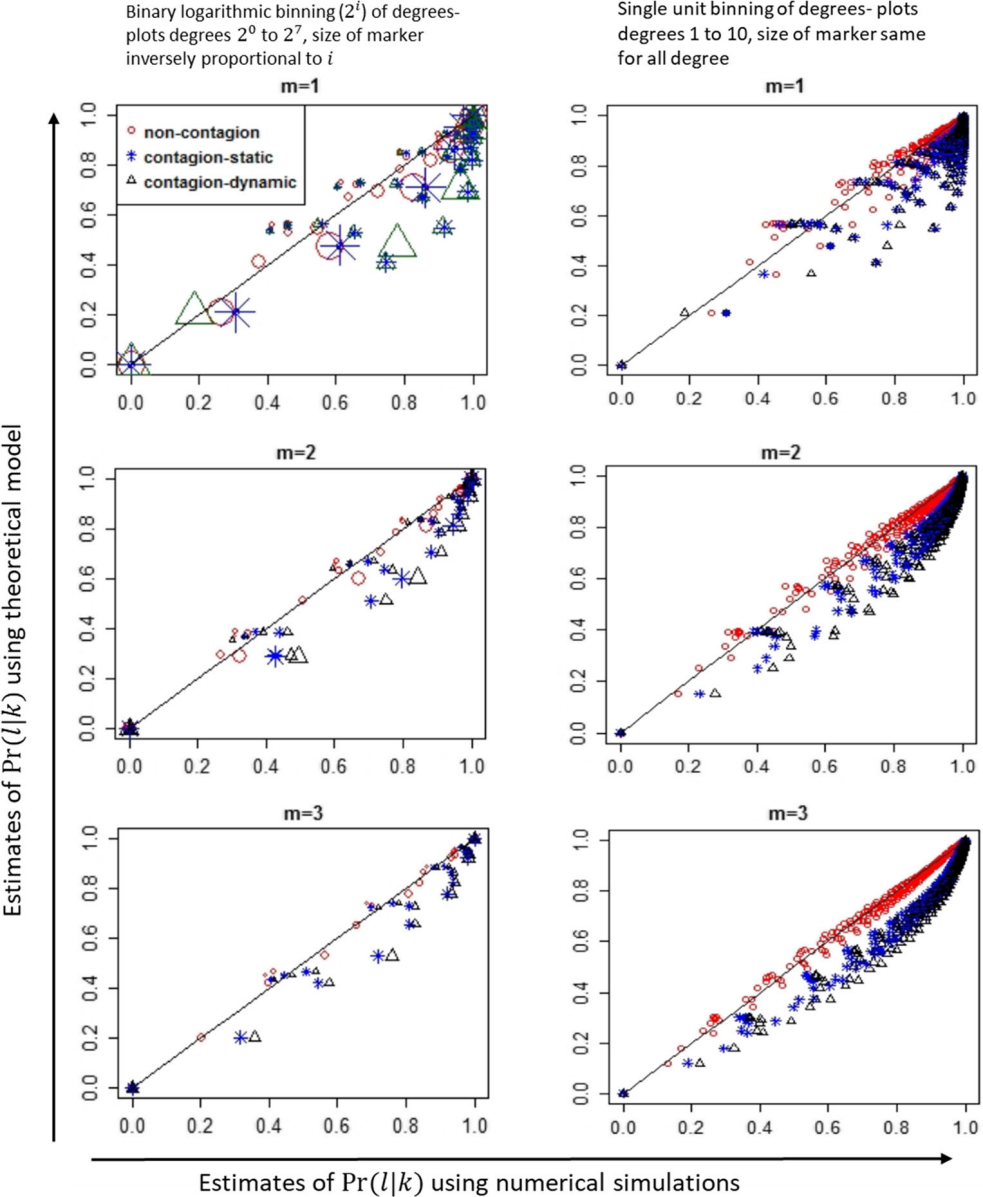
Comparing numerically estimated degree correlations on non-contagion and contagion networks with theoretically estimated distributions of degree correlations. *Pr*(*l*| *k*) is the probability that given a node of degree *k*, the degree of its neighbor is *l*. Theoretical estimates are from model in [29] (see Appendix 1c), and numerical estimates are from ABNM simulations. Results are from networks of size 1000

Figure 2 plots the conditional distributions for networks of size 1000 with different values of minimum degree and a maximum degree of 64. The characteristic feature of power law distributions is that a small number of nodes will have a large degree and most nodes have a small degree. Observing the degree distributions for the networks generated, for networks of sizes 1000 and 10,000 under different values of minimum degree, fewer than 15% of nodes have degree greater than 8 (see Appendix 4). Therefore, as typically done for scale-free networks, we first plot the conditional distributions calculated using binary logarithmic binning of degree (left-side plots of Fig. 2), and to highlight key features at lower degrees, plot conditional distributions using unit binning of degree (right-side plots of Fig. 2). Specifically, the left-side graphs of Fig. 2 plots the normalized conditional distributions as $\mathit{\Pr}\left\{{D}_A={2}^i|{D}_B={2}^j\right\}=\frac{z_{2^i\mid {2}^j}}{\sum \limits_l{z}_{2^i\mid {2}^j}},$ where ${z}_{2^i\mid {2}^j}$ is the number of occurrences of neighbors with degree in ‘bin 2^*i*^’ given degree of newly infected person is in ‘bin 2^*j*^’, where bin $$ {2}^i=\left\{\begin{array}{c}{2}^i\  if\ i\ \epsilon \left\{0,1\right\}\ \\ {}{2}^{i-1}+1\  to\ {2}^i\  if\ i>1\end{array}\right. $$. The right-hand side plots the same results with unit binning for degrees 1 to 8. There are multiple observations from this figure. First, as seen in the left-hand side subplots of Fig. 2, the theoretical estimates [29] (Appendix 1c) are a better prediction of degree correlations in scenario 1 (non-contagion network), as points lie closer to the 45 degree reference line compared to scenarios 2 (Static contagion network) and 3 (Dynamic contagion network). Second, the margin of error in scenarios 2 and 3 are inversely proportion to the degree, with degree ≤2^3^ having the largest errors. Third, degree correlations are different in static contagion networks compared to dynamic contagion networks. Results from networks of size 10,000 have similar observations (see Appendix 4).

#### Development of an evolving contact network algorithm (ECNA)

The proposed evolving contact network algorithm is outlined in Table 2. It was developed based on the findings from the previous sections that, derivation of conditional distributions of degree of neighboring nodes (*Pr*(*L* = *l*| *K* = *k*)) on ‘epidemic’ paths, could help determine the degree of the newly infected agents’ contacts. However, as currently known methods for estimating the conditional distributions are not suitable for stochastic contagion networks a new method is necessary. Considering the complexity of the stochastic process defining epidemic paths, developing an analytic expression for *Pr*(*L* = *l*| *K* = *k*) is challenging, therefore we fit a non-linear neural network model for estimations of *Pr*(*L* = *l*| *K* = *k*) as below.

**Table 2:** Evolving contact network algorithm (ECNA) (Step 4 of algorithm in Table 1)

Step 4 of Table 1: Loop through each newly infected person (*i*)	
Step 4a	Determine the number of new contacts (${\hat{d}}_i$) to generate for a newly infected person *i* as ${\hat{d}}_i=\left(\kern0.5em {d}_i-\sum \limits_j{\mathcal{A}}_t{,}_{ij}\right)$ where, ${\mathcal{A}}_t$ is the adjacency matrix of the network at time *t*, and *d*_*i*_ is the desired degree for *i*. Note that, in this method, the degree of a newly infected node *d*_*i*_ is already known at the time it becomes infected; this degree was determined in step 4b below when the node was added as a susceptible agent when one of its contacts became infected.
Step 4b	Determine *e*_*i*_, a row vector of eligible nodes from among susceptible agent nodes who are eligible to be contacts of *i*. Among persons in ${\mathcal{A}}_t$, a person *j* is eligible to be a contact of *i,* if: Constraint 1: *j* is not infected, i.e., ${h}_{\mathrm{t}-1,\mathrm{j}}=0$, where ${h}_{\mathrm{t}}$ is a row vector with ${h}_{t,i}=0$if node *i* is not infected and ${h}_{t,i}=1$ if node *i* is infected at time *t*. Constraint 2: *j* is not already a direct contact of *i*, i.e., ${\mathcal{A}}_t{,}_{ij}=0$. Constraint 3: degree of node *j* has not saturated, i.e., ${\hat{d}}_j=\left(\ {d}_j-\sum \limits_z{\mathcal{A}}_t{,}_{zj}\right)\ge$ 1. Constraint 4: 2^*l* − 1^ < *d*_*j*_ ≤ 2^*l*^; ${2}^{l-1}<{F}_{D_n\mid {d}_i}^{-1}\left(U\left[0,1\right]\right)\le {2}^l$, where ${F}_{D_n\mid {d}_i}^{-1}\left(U\left[0,1\right]\right)$ is the neighbors degree drawn from the conditional degree distribution *f*_*D* ∣ *k*_(*l*) = Pr(*D*_*n*_ = *d*_*n*_| *k*), and thus all persons who are eligible should belong to the same degree bin *l*.
Step 4c	Determine *v*_*l*_, the number of persons not in ${\mathcal{A}}_t$ who are eligible to be contacts of *i*. For persons not in ${\mathcal{A}}_t$, as described in Table 1, we only maintain a vector *v* where each element *v*_*k*_ contains the number of susceptible persons in degree bin *k*.
Step 4d	Generate contacts with $\mathit{\min}\left(\left|{e}_i\right|+{v}_l,{\hat{d}}_i\right)$ number of susceptible nodes, where |*e*_*i*_| is the size of the vector. Each new contact is randomly chosen from among the current susceptible nodes in vector *e*_*i*_ if *U*[0, 1](|*e*_*i*_| + *v*_*l*_) ≤ |*e*_*i*_|, or is newly generated to add as a susceptible node to the simulation if otherwise.

End loop

##### Non-linear neural network model for estimating degree correlations in contagion networks

By training on numerical data, we developed a neural network prediction model, which is a model-free non-linear regression for estimation of conditional distributions for degree of neighbors on epidemic paths in dynamic contagion networks. We chose neural network instead of regression because the analytical equation of the conditional probabilities are not known, and difficult to derive without the use of the adjacency matrix of the full network, which are infeasible for the proposed ABENM structure. We trained the neural network using the data generated for the numerical testing of Remarks 2 and 3 under the dynamic contagion networks in the previous section. Specifically, on the numerical data for conditional probabilities $p\left(l|k\right)=\frac{z_{k,l}}{\sum \limits_l{z}_{k,l}}$ generated using ABNM simulations, where, *z*_*k*, *l*_ was a counter in the simulation that kept track of the total number of susceptible contacts with degree *l* for newly infected persons with degree *k*, for every *k*, *l* combination. This conditional probability *p*(*l*| *k*) was set as the response variable and the following five inputs as the independent variables: the degree of the newly infected agent *k,* the degree of the susceptible neighbor of the newly infected agent *l*, the minimum degree of the network *m*, the percent of the population that is infected (to account for the changes in epidemic paths over time), and the size of the full network. The neural network was trained, in the R software, on data from 15 different scale-free networks. The data set included data from networks of size (*N*) 1000, 5000, and 10,000, and minimum network degree (*m*) of 1, 2, 3, 4, and 5, which are the inputs for generating the scale-free networks in R using *barabasi.game*, and percent infected at 5%, 13%, 25%, 35%, 50%, 60%, and 90%.

The neural net (NN) had one hidden layer and the number of hidden nodes was a hyperparameter. Tuning of the hyperparameter and validation of the NN was conducted as follows. The scale-free networks were split into test and train networks, all networks except {*N* = 1000, *m* = 1}, {*N* = 5000, *m* = 2}, and {*N* = 10000, *m* = 4}, were set as train networks. Train networks were further split into 60% and 40% train and test data, respectively, through random selection. Only train data of train networks were used in NN prediction. The hyperparameter was iteratively set to values between 5 and 14 and, under each value, the corresponding mean square error (MSE) between predicted and actual were estimated for test and train data of train networks and test networks. While the MSE decreased in the train data of train networks as the number of hidden nodes increased, on the test data and test networks, MSE first decreased and then started to increase after 8 hidden nodes (see graph in Appendix 5). Therefore, we set the NN hyperparameter value at 8 hidden nodes, and used the corresponding NN model in the ECNA. Comparison of neural network predictions with actual estimates on the test data sets are presented in Appendix 5.

When using the neural network model in ABENM, for any given value of *k*, the conditional probabilities for all values of *l* were predicted and then normalized to add to 1 prior to use in the simulation.

### Validation approach and metrics

Our validation for the newly developed simulation technique, ABENM, is to compare how well it replicates the disease predictions from an ABNM. Therefore, the key validation metric is disease prevalence (% of population infected) as a function of time, a measure of the epidemic trajectory. For ABENM simulations, we evaluated two ECNA methods that varied in their estimation of the conditional degree distributions.

- ECNA Method 1: Used the theoretical estimations for degree correlations between neighbors from [29] (see equation in Appendix 1c).
- ECNA Method 2: Used neural network predictions for degree correlations between neighbors on dynamic contagion networks

While comparing ECNA Method 2 with ABNM helps validate the newly developed algorithms, comparing with ECNA Method 1 helps understand the change in prediction errors compared to using the theoretical degree correlations. Instead of limiting our analyses to a specific disease, to evaluate the robustness of ABENM as a general alternative for studying propagation on scale-free networks, we conducted the numerical analyses for a range of assumptions representative of a wide range of diseases. Specifically, we ran ABENM (with ECNA Methods 1 and 2) and ABNM under multiple combinations of network size (*N*), network minimum degree (*m*), transmission probability per contact (*p*), initial prevalence (*i*), epidemic profiles (susceptible-infected (SI), susceptible-infected-recovered (SIR), and susceptible-infected-susceptible (SIS)), and recovery rates for SIS and SIR (*r*). Network size and minimum degree are input parameters for the preferential attachment algorithm. Minimum degree indirectly sets the scale-free network parameter (*λ*), and thus the resulting probability distribution for the number of contacts per person. Varying this feature could be representative of modeling different types of contact networks, i.e., epidemiologically different modes of transmission, e.g., sexual contacts, needle sharing contacts, or social contacts for spread of respiratory infections. Or be representative of modeling populations with different behavior, e.g., distribution of the number of sexual contacts among heterosexuals versus men who have sex with men, or distribution of social contacts in a university population versus in a general population. Static transmission probability per contact uses one value for the full simulation and is representative of the epidemiologic measure of infectiousness per contact. Varying this feature would be testing different modes of transmission for the same disease, e.g., needle sharing has a higher chance of HIV transmission than sexual contact, or testing different diseases, e.g., HIV, human papilloma virus, Hepatitis B or C all spread on same contact networks but have different infectiousness. Using a random transmission probability per contact will vary the infectiousness of an individual over time and between individuals at any time-step. Therefore, this feature could represent disease progression or intervention status that could alter infectiousness and change over time for each individual, or represent heterogeneity in individual behavior, e.g., the number of needles shared with a specific contact per unit-time. Initial prevalence could represent initial status of the epidemic at the time of analyses.

A SI epidemic profile, where persons once infected remain in that stage for the remaining duration, such as HIV, chronic Hepatitis B and C, and chronic TB, helps evaluate the growth of the full network. The training of the neural network was also conducted using SI to allow for the network to fully grow. However, we also applied the ECNA for simulating SIR and SIS epidemic profiles to evaluate the robustness of the method, specifically, the influence of interruptions in network evolution. In SIR, when an infected node recovers and becomes immune, the network growth of its uninfected contacts is altered as they can no longer be infected by the recovered node (but not necessarily terminated as they may have other contacts who could be infected). In SIS, when an infected node recovers, the alteration is similar to SIR but temporary as the recovered node can become re-infected. The rate of recovery would be the key parameter influencing the frequency of interruptions in SIR and SIS.

We specifically evaluated *N* ∈ {1000, 10000, 50000}, *m* ∈ {1, 2, .., 5}, *p* ∈ {0.01 (*static*), 0.1(*static*),  *and U*[0,0.1](*uniform random*)}, *i* ∈ {0.005,0.01,0.028} for SI. For SIR and SIS profiles, as the outbreak would die out if the overall recovery rate is higher than the infection rate, we chose a subset of combinations that sustain the epidemic for a sufficient time, specifically, *N* ∈ { 10000 }, *m* ∈ {1, 2, .., 5}, *p* ∈ {0.1 (*static*), 0.2(*static*)}, *i* ∈ {0.01}, (*r* ∈ {0.017,0.033}.

We generated 100 simulation runs for each combination. The initially infected nodes were randomly selected, i.e., for the ABNM, once the network was generated the nodes were randomly selected, and for the ABENM, the degree of the nodes generated in time-step 1 was randomly selected from the network degree-distribution. We present the 5th and 95th percentile confidence intervals as Results.

## Results

**Fig. 3:**
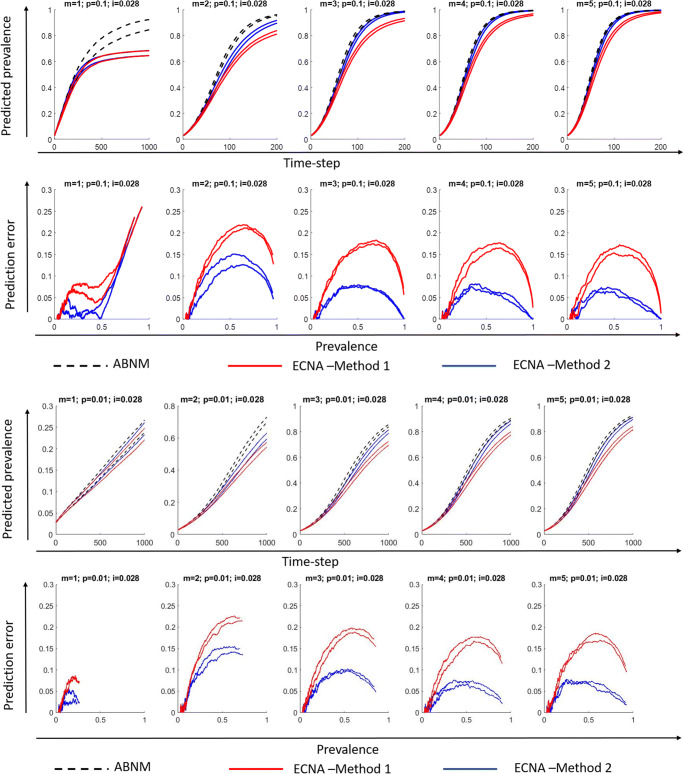
Disease prevalence (proportion of population infected) predictions and prediction errors in ABENM (ECNA Methods 1 and 2) compared to ABNM for networks with **minimum degree m = 1 to 5, transmission probability per exposure** ***p =*** **0.1 and 0.01**, **initial proportion infected i = 0.028**, and **network size** ***N*** **= 10,000**; Plots show the 5th and 95th percentile values of 100 runs**.** ABNM: Agent-based network model; ABENM: Agent-based evolving network model; ECNA- Evolving contact network algorithm; Method 1: Using theoretical estimations of degree correlations between neighbors from [29] (see Appendix 1c). Method 2: Using neural network predictions for modified degree correlations between neighbors on epidemic paths in dynamic contagion networks. (See online version in color for easier interpretation)

**Fig. 4:**
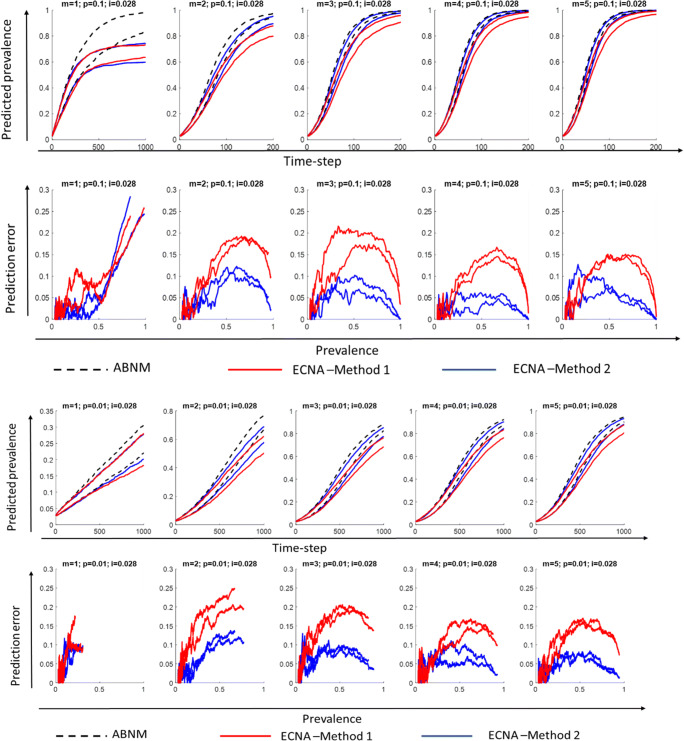
Disease prevalence (proportion of population infected) predictions and prediction errors in ECNA Methods 1 and 2 compared to ABNM, for networks with **minimum degree m = 1 to 5, transmission probability per exposure** ***p =*** **0.1 and 0.01, initial proportion infected i = 0.028, and network size** ***N*** **= 1000**; Plots show the 5th and 95th percentile values of 100 runs**.** ABNM: Agent-based network model; ABENM: Agent-based evolving network model; ECNA- Evolving contact network algorithm; Method 1: Using theoretical estimations of degree correlations between neighbors from [29] (see Appendix 1c). Method 2: Using neural network predictions for modified degree correlations between neighbors on epidemic paths in dynamic contagion networks. (See online version in color for easier interpretation)

We first discuss results for SI. Results for disease prevalence (% of population infected), specifically the 5th and 95th percentile ranges of the 100 runs on networks with varying combinations of minimum degree (1 to 5) and probability of transmission per contact (0.1 or 0.01), keeping initial prevalence at 0.028 are presented in Figs. 3 and 4 for networks of sizes 10,000 and 1000, respectively. It is observed that Method 2 outperforms Method 1 in most scenarios as hypothesized, i.e., the 5th and 95th percentile values of the 100 runs overlap more closely in ECNA Method 2 than ECNA Method 1 in most cases. The deviations in the 5th and 95th percentile values in ABENM compared to ABNM, calculated as the absolute difference in values between ABNM and ABENM divided by ABNM, are presented as prediction errors. Prediction errors are below 15% in ECNA Method 2 in all cases except when minimum degree was 1. In the networks with minimum degree of 1, the errors are less than 10% up until 50% prevalence. The prediction errors decrease as the minimum degree increases. The probability of transmission had little effect on the results. While the prediction errors fluctuated considerably in population size 1000, especially at the initial phase of the epidemic, it was more stable when the population size was 10,000. Results under different combinations of minimum degree (1 to 5) and probability of transmission per contact (0.1 and 0.01), for lower values of initial prevalence (0.01 and 0.005) are presented in Appendix 5 and 6 for networks of sizes 10,000 and 1000, respectively. For these lower values of initial prevalence, and for networks of size 10,000, the prediction errors were similar to above in all cases except when minimum degree was 1 where the errors were unacceptably high. For networks of size 1000, as the value of initial prevalence decreased, the errors in the initial phase of the infection increased. Results using random transmission probability per contact are presented in Appendix 7 for networks with different values of minimum degree (1 to 5), and keeping initial prevalence at 0.01 and network size at 10,000. Results for random transmission probability per contact on networks with minimum degree 2, initial prevalence at 0.005, and network size 50,000 are also presented in Appendix 7. Results on these networks were similar as in static transmission probability per contact, i.e., prediction errors were at most 15% when minimum degree was 2 or higher.

The prediction errors in ECNA Method 2 for epidemic profiles SIR and SIS followed similar trends in the combinations evaluated as the SI profile, however, errors were higher than in SI profile. Errors were 20% or below except when the epidemic died out quickly, specifically when minimum degree was 1 and in some instances of minimum degree 2 (Appendix 9 and 10). However, errors in ECNA Method 2 were generally lower than in ECNA Method 1 (Appendix 9 and 10).

## Discussion and conclusions

This is a concept paper to present a new simulation modeling technique, ABENM, which combines theories from compartmental and agent-based modeling, commonly used simulation techniques in disease epidemic predictions. The motivation for this new simulation technique is to provide a computationally feasible alternative to ABNM, which is necessary for analysis of interventions related to low prevalence diseases. Though the ABENM framework is directly based on theories from two well-known concepts, compartmental modeling and ABNM, we believe this is the first paper to propose a hybrid model and present a structure for such a hybrid simulation technique. Further, a significant contribution is the development of a new evolving contact network algorithm (ECNA), for generating scale-free networks without generating the full network, which is key to enabling such a hybrid simulation method.

The key contributions of this work can be summarized as follows. It presents a new agent-based network simulation modeling technique that simulates only infected persons and their network of immediate contacts at the individual-level. While the concept is simple, generating partial networks and evolving them over time as new persons become infected is challenging, and our work identifies key concepts to help further research in this area. Our work extends beyond what is already known about degree correlations as a significant structural property of scale-free networks for studying diffusion in networks. Specifically, our work suggests that the degree correlations while traversing through epidemic paths of the network are different than when generally evaluating the degree correlation of the full network, as shown in numerical simulations (also see Remarks 2 and 3 in Appendix 3), due to the influence of the stochastic process defining the epidemic. Further, considerably lower epidemic prediction errors in Method 2 over Method 1 suggests that the ECNA, that combines simple concepts from graph theory with neural network function approximations, is a good approach for generating an evolving contact network in ABENM.

The empirical results also identify the cases under which the proposed ABENM would be suitable, and help inform development of simulation models for diseases. First, the prediction errors for SI epidemic profiles were below 15% for networks of size 10,000 in all cases except when minimum network degree was 1. This implies that, in modeling say an HIV epidemic, persons with 1 lifetime partners should not be included. As persons with degree of 1 are at the end of a path, their contribution to disease transmission is insignificant, and thus exclusion of those with degree 1 or 0 will likely have minimal impact on epidemic predictions.

Second, while the networks of size 10,000 and 50,000 had prediction errors below 15%, the smaller network size of 1000 had larger prediction errors and were less stable in the initial phase of the simulation, highlighting the significance of sample size. This implies that ABENM is not suitable for small networks, and moreover, not necessary as small networks can be analyzed using ABNM.

Third, for networks of size 10,000, while predictions errors for SI were at most 15% in all cases (excluding minimum degree 1 discussed above), they were below 10% when minimum degree was greater than 2. When minimum degree was 2, prediction errors were below 10% up until prevalence of 10%. In networks of size 50,000, when minimum degree was 2, prediction errors were below 10% until the end of simulations which were terminated at 50% prevalence. This implies that larger network sizes should be chosen, which is suitable as the motivation for ABENM is to enable simulation of large population sizes to maintain a good sample size for modeling heterogeneity.

Fourth, prediction errors were generally higher in SIR and SIS compared to SI, which is as expected as the neural network was trained only with SI profiles. More specific neural network training could help improve the prediction including the use of additional independent variables such as proportion recovered. Prediction errors were however lower in ECNA Method 2 compared to Method 1, providing more stability across the different epidemic profiles. The interpretation of these prediction errors is that, 95% of the time the prediction errors will be lower than that noted above. Therefore, for determining whether to use ABENM for any specific application, the acceptability of the above margin of errors should be considered and subsequent results interpreted accordingly.

We believe the significance of the contributions from this work is in the study of diseases where contact structures are critical that compartmental models are not a suitable option and have low prevalence that agent-based network models are infeasible to use. Though our analysis here were restricted to populations of size below 50,000, the lower prediction errors with higher population size is encouraging because the expected use for ABENM is for simulating diseases with small prevalence but with larger population sizes. Instead of scaling-down the full population as in ABNM, ABENM will only scale the infected population. For example, for simulating a prevalence say upto 5% (50,000 infected persons) in a population of 1 million, ABNM would first generate a smaller scaled version of the 1 million (say 10,000) and simulate this network until 5% (i.e., 500 persons) become infected. On the other hand, when using ABENM, we could simulate upto 50,000 infected persons or scale it down while maintaining a sufficiently large sample, say 10,000 infected persons.

This work is subject to limitations. The ABENM presented here would be suitable for only those networks that follow scale-free property. We tested the validity of ABENM on a simple version of the model, only indirectly testing impact of disease progression and behavior, and should be followed with a more specific application to diseases. One such study extended this work to model HIV in the United States, by modeling comprehensive dynamics and heterogeneity in population demographics and risk behavior, and showed good validation with national surveillance data on multiple epidemic and network features. [30, 31] The results from the analysis conducted here on hypothetical networks and data assumptions, representative of a wide range of disease and behavioral features, are promising for application of the ABENM as an alternative simulation technique for study of other diseases with low prevalence.

In this manuscript, we used neural networks to predict assortative correlations in degree of neighboring nodes under the influence of a stochastic process (disease transmissions). The scope of work was limited to scale-free networks where only one network feature influences degree correlations between neighbors, the scale-free network exponent (*λ*). Future work could expand this to networks of other types that also have degree correlations, if an ABENM simulation on that network type is desirable. Other types of degree correlations include second neighbor degree correlations or clustering that are typical in Watts-Strogatz small-world networks used for modeling social friendship networks. [32] In such networks, two friends of a node have a higher than random chance of also being friends with each other. Thus, the probability of forming triangles between any three nodes, modeled using clustering coefficient, would be a relevant feature and should be incorporated as an additional independent variable in the training of a neural network. Similarly, networks of other types should be trained separately using the features that influence degree correlations, modeling them as independent variables of the neural network model.

In the context of diseases, there may be other forms of associative correlations, i.e., the nature of humans to associate with persons who are similar to them, commonly known as homophily, that maybe relevant. These features, however, may not necessarily influence degree correlations, and therefore may need to be considered outside the structure of neural networks. For example, for sexually transmitted diseases, age of partners (age of neighboring nodes) are relevant, as persons of similar age are more likely to form partnerships, but it does not impact degree correlation when modeling lifetime partners as done by one HIV model [30, 31]. They used the neural network presented here to predict the number of lifetime number of partners (degree) of a neighboring node, but developed new optimization methods to model the current age of the neighbor, the age at which the partnership would initiate, and the duration of the partnership, considering all of its lifetime partnerships.

We believe, this paper helps establish that degree correlations is a key feature impacting the scale-free network structure under the influence of an epidemic, all other stochastic features including behavioral and epidemic features, while relevant for the disease dynamics, are not significant for the network structure. This conclusion can be drawn from the fact that the different probabilities of transmission, including static and dynamic values that varied over a range that included zero, had no impact on the results though they were not included as an independent variable in the training of the neural network. Therefore, once trained on scale-free networks using the power-law distribution exponent (*λ*) and proportion infected as the independent variables, the trained neural network can be used in an ABENM to simulate different populations and epidemics, on a scale-free contact network structure of any value of *λ*. Stochastic changes in behavior as people age, changes in partnership as persons age, or changes in disease features can be simulated specific to a disease and population.

The numerical analyses conducted in this paper indicates that the method is applicable for diseases and analysis of interventions that change the exposure or risk to infection over time, and interventions that activate or deactivate the status of a link. Taking HIV as an example, interventions that change exposure to risk and thus interventions that can be modeled include early diagnosis of HIV infected persons, which is known to increase condom use behavior and thus transmission risk, linkage to care and treatment of HIV infected persons, which is known to reduce transmission risk, interventions that alter condom use behavior, and pre-exposure prophylaxis for susceptible/uninfected persons, which reduce their risk of acquisition. Interventions that activate or deactivate the status of a link include needle exchange programs for injecting drug users, which alters needle sharing behavior. Transmission risk can be modeled as a function of the behavioral parameters of the nodes involved in the partnership, as commonly done. A susceptible person who is a contact of an infected person in the network can be modeled to have prophylaxis or vaccination status, and use it in the transmission risk function. Prophylaxis or vaccination status among susceptible persons in the compartmental model can be modeled as a heterogeneous parameter by splitting compartments into vaccinated and not vaccinated and applying certain rates of transitioning from one to the other. Changes in needle sharing can be modeled by activating or deactivating links using the dynamic adjacency matrix of the ABENM. Similarly, the impact of interventions that may reduce the number of contacts can be measured by activating links in a no intervention scenario and deactivating them in an intervention scenario through the use of the dynamic adjacency matrix in ABENM. On the other hand, if there are systematic changes that permanently alter the degree distribution for future generations, then that transition should be given special focus and additional analysis should be conducted depending on the type of transition. For example, if it still maintains a scale-free network structure but the value of the power-law exponent (*λ*) is altered permanently, the neural network need not be retrained, the model can be set to draw for this new *λ*, but the transitionary phase might be relevant to analyze further and be specific to the problem studied. Interventions that permanently change the network structure such that it is no more scale-free, are outside the scope of this model.

In summary, we believe the proposed ABENM contributes to the simulation modeling literature by serving as an alternate technique to the current extreme techniques of agent-based and compartmental simulation modeling. The ECNA contributes to the network generation algorithm literature for simulation of epidemic projections over scale-free contact networks. Future work could explore development of network generation algorithms for other types of networks using a neural network method as that developed here.

## Supplementary Information

ESM 1 (PDF 4898 kb)
