# Supplementary material for: Agent-based evolving network modeling: a new simulation method for modeling low prevalence infectious diseases
Source: Health Care Manag Sci. 2021 May 15;24(3):623–39. doi: 10.1007/s10729-021-09558-0 (PMC8459606; doi:10.1007/s10729-021-09558-0)
Supplement: Supplementary file 1 — (PDF 4898 kb) [file 10729_2021_9558_MOESM1_ESM.pdf]

Online Appendix

Agent-based evolving network modeling: a new simulation method for modeling low prevalence infectious diseases

Contents

Appendix I: Computational modeling methods 2

Appendix Ia: General structure of compartmental model 2

Appendix Ib: Comparing and deriving ABENM from ABNM 3

Appendix Ic: Previous work in analytical models of degree correlations in scale-free networks 6

Appendix II: Extension of the ABENM to other disease structures 7

Appendix IIa: Extension of the ABENM to SIIR disease structures 7

Appendix IIb: Extension of the ABENM SIR disease structure to include heterogeneity 8

Appendix IIc: Extension of the ABENM structure to model births and deaths........ 9

Appendix III: Degree correlations between neighbor nodes on epidemic paths in networks 12

Appendix IV: Scale-free network distributions 15

Appendix V: Neural network predictions for degree correlation on test networks........ 18

Appendix VI: Sensitivity analysis - Results for epidemic predictions under varying values of minimum degree, transmission probability, and initial infection on networks of size 10,000 (Susceptible-Infected (SI) epidemic) 20

Appendix VII: Sensitivity analysis - Results for epidemic predictions under varying values of minimum degree, transmission probability, and initial infection on networks of size 1000 (Susceptible-Infected (SI) epidemic) 23

Appendix IX: Sensitivity analysis – Susceptible-Infected-Recovered (SIR) epidemic........ 27

Appendix X: Sensitivity analysis – Susceptible-Infected-Susceptible (SIS) epidemic........ 29

Appendix I: Computational modeling methods

Appendix Ia: General structure of compartmental model

A compartmental model for simulating the epidemic trajectory can be represented as a continuous time non-stationary Markov process $X = \{X_t;\ t \geq 0, \Omega,\ Q_t,\ \rho_t\}$, where, X_t is the disease state of an individual defined over the state space Ω, the non-stationary transition rate matrix Q_t, and the state distribution ρ_t at time t. Taking the simplest epidemic structure Susceptible-Infected-Removed (SIR) (Removed usually represents either recovery or mortality, in the case of HIV this represents mortality) as an example, $\Omega = \{\mathrm{S}, \mathrm{I}, \mathrm{R}\}$, and $\rho_t = [s_t \quad i_t \quad r_t]$, and s_t, i_t, r_t= proportion of people in states S, I, and R, respectively, at time t.
We can represent the transition rate matrix as:

$$Q_t = \begin{array}{c} \\ S \\ I \\ R \end{array} \begin{array}{c} \begin{array}{ccc} S & I & R \end{array} \\ \begin{bmatrix} -pc_t i_t - \mu_S & pc_t i_t & \mu_S \\ 0 & -\mu_I & \mu_I \\ 0 & 0 & 0 \end{bmatrix} \end{array}$$

where,
p = probability of transmission per susceptible-infected contact
c_t = average number of contacts per person at time t
$c_t i_t$= average number of infected contacts per person at time t, because of the assumption of uniform mixing in compartmental model the proportion of contacts who are infected is simply the proportion infected in the population (i_t)(in the case of HIV, as R represents death, this equation would be $\frac{c_t i_t}{N - r_t}$ as there can be no contacts with persons in R)
μ_S = rate of transitioning from state S to R (in the case of HIV it represents natural mortality rate)
μ_I = rate of transitioning from state I to R (in the case of HIV it represents mortality rate from the disease)

<u>Epidemic trajectory predictions: estimations of s_t, i_t, r_t, over time t</u>
The trajectory of the epidemic, defined by projections of s_t, i_t, and r_t, over time t, can be numerically determined by iteratively solving a system of differential equations, iterating over time t, with a sufficiently small time-step Δt, as

$$\rho_t = \rho_{t-1} + \frac{dQ_{t-1}}{dt}\Delta t$$

where,

$$\frac{dQ_{t-1}}{dt} = \rho_{t-1}Q_{t-1}$$

Specifically, expanding the above, we can write

$$s_t = s_{t-1} - s_{t-1}pc_{t-1}i_{t-1} - s_{t-1}\mu_S \quad (1)$$
$$i_t = i_{t-1} + s_{t-1}pc_{t-1}i_{t-1} - i_{t-1}\mu_I \quad (2)$$
$$r_t = r_{t-1} + s_{t-1}\mu_S + i_{t-1}\mu_I \quad (3)$$

Without loss of generality, and as typically done in compartmental model, instead of using the Markov processes representation of s_t, i_t, r_t as the proportion of people in states S, I, and R, respectively, where $s_t + i_t + r_t = 1$, we can rewrite these equations using S_t, I_t, R_t as the number of people in states S, I, and R, respectively, with $S_t + I_t + R_t = N$, where N is the size of total population.

Appendix Ib: Comparing and deriving ABENM from ABNM

ABNM framework

In agent-based network modeling (ABNM), features related to individuals can be tracked using the following parameters.

$\mathbb{A}$ = an adjacency matrix of size NXN with binary elements $\mathbb{A}_{ij}$ i.e., $\mathbb{A}_{ij} = 1$ if nodes i and j are contacts, and 0 otherwise, with N = the number of people in the population. We make $\mathbb{A}$ static to represent long-term contacts. For example, for HIV, $\sum_j \mathbb{A}_{ij}$ would represent the number of lifetime partnerships of person i.
$\mathbb{V}_t$: $\mathbb{V}_{t,ij} \leq \mathbb{A}_{ij}$= a matrix of size NXN that tracks if contacts are active or inactive, i.e., $\mathbb{V}_{t,ij} = 1$ if contacts between i and j are active at time t, and 0 otherwise. We make $\mathbb{V}_t$ dynamic so as to model the dynamic changes in contacts, e.g., $\mathbb{V}_{t,ij} = 1$, if i and j are contacts and there was needle sharing at time-step t, 0 otherwise.
$\mathbb{h}_t$ = a row vector of size N with each element j taking a binary value, 1 if person j is infected and 0 otherwise, and dynamically changing with time t,
$\mathbb{m}_t$ = a row vector of size N with each element j taking a binary value, 1 if person j is deceased and 0 otherwise, and dynamically changing with time t,
$\mathbb{c}_t$ = a row vector of size N with value of element j equal to the number of active infected contacts of person j if j is susceptible and alive and zero otherwise, and dynamically changing with time t,
$\mathbb{u}$ = a unit row vector of size N

ABENM framework:

In the proposed agent-based evolving network modeling ABENM, we keep track of only infected persons and their immediate contacts at the individual-level, using the following parameters.

$\mathcal{A}_t$ = a static adjacency matrix with dynamically changing size $Q_t \times Q_t$, where Q_t is the number of people modeled at the individual-level (i.e., only infected persons and their immediate contacts) at time t, and representing long-term contacts equivalent of $\mathbb{A}$. That is, in a fully connected network, and in the limit that all persons become eventually infected, $\mathcal{A}_t \rightarrow \mathbb{A}$ as $Q_t \rightarrow N$,
V_t = a dynamic adjacency matrix of dynamically changing size $Q_t \times Q_t$, equivalent of $\mathbb{V}_t$ to model dynamic changes in contacts,
$\mathcal{h}_t$ = a row vector of size Q_t with each element j taking a binary value, 1 if person j is infected and 0 otherwise,
$\mathcal{m}_t$ = a row vector of size Q_t with each element j taking a binary value, of 1 if person j is deceased and 0 otherwise,
c_t = a row vector of size N with the value of element j equal to the number of active infected contacts of person j if j is susceptible and alive and zero otherwise
$\mathcal{u}_t$ = a unit row vector of size Q_t, and
$F^{-1}(a)$ = inverse Bernoulli distribution that takes values 1 with probability a and 0 with probability $1 - a$.

Epidemic trajectory projections using ABENM

Remark 1: For an SIR epidemic, the epidemic trajectory projections using ABENM can be modeled using a hybrid compartmental and ABNM structure as below.

$$s_t = s_{t-1} - \frac{\sum_{j=1:Q_{t-1}} F^{-1}(1-(1-p)^{c_{t-1,j}})}{\mathrm{N}} - s_{t-1}\mu_S$$

$$i_t = \frac{\hbar_{t-1}u_t^T + \sum_{j=1:Q_{t-1}} F^{-1}(1-(1-p)^{c_{t-1,j}}) - \mu_I \hbar_{t-1}u_t^T}{N}$$

$$r_t = r_{t-1} + s_{t-1}\mu_S + \frac{\mu_I \hbar_{t-1}u_t^T}{N}$$

Proof: Using the ABNM framework above, we can extract the following key epidemic features of the network by applying elementary matrix operations:

- A row vector of size N with non-zero values corresponding to indices of only susceptible persons $= (\mathbb{u} - \mathbb{h}_{t-1}) \circ (\mathbb{u} - \mathbb{m}_{t-1})$, where $\circ$ is element wise multiplication
- The number of susceptible persons $= (\mathbb{u} - \mathbb{h}_{t-1})(\mathbb{u} - \mathbb{m}_{t-1})^T$ (T represents transpose)
- The proportion of susceptible persons $= s_{t-1} = \frac{(\mathbb{u} - \mathbb{h}_{t-1})(\mathbb{u} - \mathbb{m}_{t-1})^T}{N}$
- The proportion of infected persons $= i_{t-1} = \frac{\mathbb{h}_{t-1}\mathbb{u}^T}{N}$
- The proportion of removed persons $= r_{t-1} = \frac{\mathbb{m}_{t-1}\mathbb{u}^T}{N}$
- A row vector with each element j equal to the number of infected contacts of $j = (\mathbb{A}\mathbb{h}_{t-1}^T)^T$
- A row vector of size N with each element j the number of active infected contacts at time t as $\mathbb{c}_t = \left((\mathbb{u} - \mathbb{h}_t) \circ (\mathbb{u} - \mathbb{m}_t) \circ ((\mathbb{A}\mathbb{h}_t^T)^T)\right)$, i.e., $\mathbb{c}_{t,j}$ is the number of infected contacts of node j if j is susceptible and alive, and $\mathbb{c}_{t,j}$ is zero otherwise.
- Note: For the case of dynamically changing contacts all instances of $\mathbb{A}$ should be multiplied by $\mathbb{V}_{t-1}$, e.g., the row vector representing the number of infected contacts would be written as $= \left((\mathbb{A} \circ \mathbb{V}_{t-1})\mathbb{h}_{t-1}^T\right)^T$. For purposes of clarity, we write this as $(\mathbb{A}\mathbb{h}_{t-1}^T)^T$, and note that for dynamic contacts all instances of $\mathbb{A}$ should be multiplied by $\mathbb{V}_{t-1}$.

Then, instead of the compartmental modeling structure that estimates the proportion of population who are new infections as $s_{t-1}pc_{t-1}i_{t-1}$, which is derived assuming an average number of contacts per person as c_{t-1}, multiplying it by i_{t-1} to get an average number of contacts who are infected, multiplying it by p to get an average number of new infections per susceptible person, and finally multiplying it by the proportion of susceptible persons s_{t-1} to get the proportion of population who are new infections, we can use individual-level contact structures to estimate the proportion of population who are new infections as

$$(ps_{t-1}c_{t-1}i_{t-1}) = \frac{(\mathbb{u} - \mathbb{h}_{t-1})(\mathbb{u} - \mathbb{m}_{t-1})^T}{N} \; \frac{\sum_{j=1:N} F^{-1}(1-(1-p)^{\mathbb{c}_{t-1,j}})}{(\mathbb{u} - \mathbb{h}_{t-1})(\mathbb{u} - \mathbb{m}_{t-1})^T}$$

which is obtained by replacing the compartmental modeling equations with the equivalent ABNM equations from above, specifically,

proportion of susceptible persons, $s_{t-1} = \frac{(\mathbb{u} - \mathbb{h}_{t-1})(\mathbb{u} - \mathbb{m}_{t-1})^T}{N}$,

number of infected contacts per susceptible person, $c_{t-1}i_{t-1} = \frac{\sum_{j=1:N} \mathbb{c}_{t-1,j}}{(\mathbb{u} - \mathbb{h}_{t-1})(\mathbb{u} - \mathbb{m}_{t-1})^T}$, and

new infections per susceptible person, $pc_{t-1}i_{t-1} = \frac{\sum_{j=1:N} F^{-1}\left(1-(1-p)^{\mathbb{c}_{t-1,j}}\right)}{(\mathbb{u} - \mathbb{h}_{t-1})(\mathbb{u} - \mathbb{m}_{t-1})^T}$, which uses the more accurate individual-level Bernoulli equation $F^{-1}(1-(1-p)^{\mathbb{c}_{t-1,j}})$ to determine the transmission per person, summing over N individuals to determine the number of transmissions, and dividing by the number of susceptible persons to determine new infections per susceptible person.

Using the above ABNM derivations for i_{t-1} and $ps_{t-1}c_{t-1}i_{t-1}$ in the compartmental epidemic trajectory projection model in (1), (2), and (3) of the main manuscript will result in the following hybrid epidemic prediction model.

$$s_t = s_{t-1} - \frac{\sum_{j=1:N} F^{-1}(1-(1-p)^{\mathbb{c}_{t-1,j}})}{N} - s_{t-1}\mu_S$$

$$i_t = \frac{\mathbb{h}_{t-1}\mathbb{u}^T + \sum_{j=1:N} F^{-1}(1-(1-p)^{\mathbb{c}_{t-1,j}}) - \mu_I \mathbb{h}_{t-1}\mathbb{u}^T}{N}$$

$$r_t = r_{t-1} + s_{t-1}\mu_S + \frac{\mu_I \mathbb{h}_{t-1}\mathbb{u}^T}{N}$$

That is, the aggregated estimations for proportion infected (i_{t-1}) and proportion newly infected ($ps_{t-1}c_{t-1}i_{t-1}$) of compartmental model in (1), (2) and (3) of main manuscript is replaced with the individual-level estimations from ABNM, while all other parameters are maintained as in compartmental modeling. We can replace $\mathbb{m}_{t-1}$, $\mathbb{h}_{t-1}$, $\mathbb{c}_{t-1}$ and $\mathbb{A}$ with $\mathcal{m}_{t-1}$, $\mathcal{h}_{t-1}$, c_{t-1}, and $\mathcal{A}_{t-1}$, respectively, as all elements of $\mathbb{m}_{t-1}$, $\mathbb{h}_{t-1}$, and $\mathbb{A}$ that are not in $\mathcal{m}_{t-1}$, $\mathcal{h}_{t-1}$, and $\mathcal{A}_{t-1}$represent susceptible persons who cannot transmit infection, and thus, the values of their corresponding terms in the above equations are 0.

$$s_t = s_{t-1} - \frac{\sum_{j=1:Q_{t-1}} F^{-1}(1-(1-p)^{c_{t-1,j}})}{N} - s_{t-1}\mu_S$$

$$i_t = \frac{\mathcal{h}_{t-1}\mathcal{u}_t^T + \sum_{j=1:Q_{t-1}} F^{-1}(1-(1-p)^{c_{t-1,j}}) - \mu_I \mathcal{h}_{t-1}\mathcal{u}_t^T}{N}$$

$$r_t = r_{t-1} + s_{t-1}\mu_S + \frac{\mu_I \mathcal{h}_{t-1}\mathcal{u}_t^T}{N}$$

Thus, while infected persons and immediate contacts are tracked at the individual-level as in ABNM using $\mathcal{m}_{t-1}$, $\mathcal{h}_{t-1}$, and $\mathcal{A}_{t-1}$, all other susceptible and removed persons are tracked at the aggregated-level as in compartmental model using s_{t-1} and r_{t-1}.
This completes the proof.

Appendix Ic: Previous work in analytical models of degree correlations in scale-free networks

Analytical model for estimating degree correlations in general (non-contagion) scale-free networks:

Fotouhi and Rabbat, 2013, present an analytical model for the conditional distribution $Pr(L = l|k)$ derived generally for scale-free networks [29]. This model is based on the theoretical degree correlation at steady state, i.e., for a fully developed network. The probability mass function is given as:

$$f_{L|k}(l) = Pr(L = l|k) = p(l|k) = \frac{m(k+2)}{kl(l+1)} - \frac{m}{kl} B_{m+1}^{2m+2} \frac{B_{l-m}^{k+l-2m}}{B_{l}^{k+l+2}}$$

where,

B_y^x denotes the binomial coefficient $\binom{x}{y}$,

m is the minimum degree of the network, and

k is the degree of the newly infected node.

However, this model does not consider the underlying stochastic process representing epidemic trajectories, as discussed next.

Appendix II: Extension of the ABENM to other disease structures

Appendix IIa: Extension of the ABENM to SIIR disease structures

Susceptible(S)-Infected and Latent ($\bar{I}$)-Infectious(I)-Removed(R):

Let,

$\mathcal{A}_t$ be a static adjacency matrix of size $Q_t \times Q_t$, where Q_t is the number of people to model at the individual-level, say persons in $\bar{I}$, I, and R and their immediate contacts.

V_t be a dynamic adjacency matrix of size $Q_t \times Q_t$

$\bar{\hbar}_t$ be a row vector of size Q_t taking binary values, 1 if a person is in $\bar{I}$ and 0 otherwise,

$\hbar_t$ be a row vector of size Q_t taking binary values, 1 if a person is in I and 0 otherwise,

$\mathcal{m}_t$ be a row vector of size Q_t taking binary values, 1 if a person is in R and 0 otherwise,

$\mathcal{u}_t$ be a unit array of size Q_t

$\gamma_{\bar{I}}$ be the rate of transitioning from $\bar{I}$ to I

$\mu_{\bar{I}}$ be the rate of transitioning from $\bar{I}$ to R

μ_I be the rate of transitioning from I to R

N is the population size

c_t is a row vector, with $c_{t,j}$ = the number of infected contacts of j if j is susceptible and alive, and = 0 otherwise, and given by $c_t = (\mathcal{u} - \hbar_{t-1}) \circ (\mathcal{u} - \mathcal{m}_{t-1}) \circ \left((\mathcal{A}_{t-1} \circ V_{t-1})\, \hbar_{t-1}^T \right)^T$

then, in the ABENM structure, epidemic predictions over time t, i.e., the proportion of persons in each stage can be calculated as

$$s_t = s_{t-1} - \frac{\sum_{j=1:Q_{t-1}} F^{-1}\left(1-(1-p)^{c_{t-1,j}}\right)}{N} - s_{t-1}\mu_S$$

$$\bar{\imath}_t = \frac{\bar{\hbar}_{t-1}\mathcal{u}_{t-1}^T + \sum_{j=1:Q_{t-1}} F^{-1}(1-(1-p)^{c_{t-1,j}}) - \gamma_{\bar{I}}\bar{\hbar}_{t-1}\mathcal{u}_{t-1}^T - \mu_{\bar{I}}\bar{\hbar}_{t-1}\mathcal{u}_{t-1}^T}{N}$$

$$i_t = \frac{\gamma_{\bar{I}}\bar{\hbar}_{t-1}\mathcal{u}_{t-1}^T - \mu_I \hbar_{t-1}\mathcal{u}_{t-1}^T}{N}$$

$$r_t = r_{t-1} + s_{t-1}\mu_S + \frac{\mu_I\bar{\hbar}_{t-1}\mathcal{u}_{t-1}^T}{N} + \frac{\mu_I\hbar_{t-1}\mathcal{u}_{t-1}^T}{N}$$

Compared to the SIR structure, the changes in the SIIR structure are the addition of an equation to represent the new stage, using $\hbar_{t-1}$ and $\bar{\hbar}_{t-1}$ to separate persons who are infectious from those infected but not infectious, such that only $\hbar_t$ is used in the transmission equation, and addition of transition rates specific to the additional stages. Without loss of generality, we can conclude that the ABENM structure can be applied to epidemics of different structures.

Appendix IIb: Extension of the ABENM SIR disease structure to include heterogeneity

Susceptible(S)-Infectious(I)-Removed(R) and Susceptible($\bar{S}$)-Infectious($\bar{I}$)-Removed($\bar{R}$) represents a SIR disease structure with the population split into two heterogeneous groups: Let,

$\mathcal{A}_t$ be a static adjacency matrix of size $Q_t \times Q_t$, where Q_t is the number of people to model at the individual-level, say persons in I, R, $\bar{I}$, and $\bar{R}$ and their immediate contacts, mixing between population groups will be modeled through this matrix.

V_t be a dynamic adjacency matrix of size $Q_t \times Q_t$

$\hbar_t$ be a row vector of size Q_t taking binary values, 1 if a person is in I and 0 otherwise,

$\bar{\hbar}_t$ be a row vector of size Q_t taking binary values, 1 if a person is in $\bar{I}$ and 0 otherwise,

m_t be a row vector of size Q_t taking binary values, 1 if a person is in R and 0 otherwise,

$\bar{m}_t$ be a row vector of size Q_t taking binary values, 1 if a person is in $\bar{R}$ and 0 otherwise,

u_t be a unit array of size Q_t

μ_I be the rate of transitioning from I to R

$\mu_{\bar{I}}$ be the rate of transitioning from $\bar{I}$ to $\bar{R}$

N is the population size

c_t is a row vector, with $c_{t,j}$ = the number of infected contacts of j if j is susceptible and alive, and = 0 otherwise, and given by $c_t = \left(u_t - (\hbar_t + \bar{\hbar}_t)\right) \circ (u_t - m_t) \circ \left(\mathcal{A}_t(\hbar_t + \bar{\hbar}_t)^T\right)^T$

Then, in the ABENM structure, epidemic predictions over time t, i.e., the proportion of persons in each stage can be calculated as

$$s_t = s_{t-1} - \frac{\sum_{j=1:Q_{t-1}}\{F^{-1}(1-(1-p)^{c_{t-1,j}}) \circ \hbar_{t-1}\}}{N} - s_{t-1}\mu_S$$

$$i_t = \frac{\hbar_{t-1}u_{t-1}^T + \sum_{j=1:Q_{t-1}}\{F^{-1}(1-(1-p)^{c_{t-1,j}}) \circ \hbar_{t-1}\} - \mu_I \hbar_{t-1}u_{t-1}^T}{N}$$

$$r_t = r_{t-1} + s_{t-1}\mu_S + \frac{\mu_I \hbar_{t-1}u_{t-1}^T}{N}$$

$$\bar{s}_t = \bar{s}_{t-1} - \frac{\sum_{j=1:Q_{t-1}}\{F^{-1}(1-(1-p)^{c_{t-1,j}}) \circ \bar{\hbar}_{t-1}\}}{N} - \bar{s}_{t-1}\mu_{\bar{S}}$$

$$\bar{\iota}_t = \frac{\bar{\hbar}_{t-1}u_{t-1}^T + \sum_{j=1:Q_{t-1}}\{F^{-1}(1-(1-p)^{c_{t-1,j}}) \circ \bar{\hbar}_{t-1}\} - \mu_{\bar{I}} \bar{\hbar}_{t-1}u_{t-1}^T}{N}$$

$$\bar{r}_t = \bar{r}_{t-1} + \bar{s}_{t-1}\mu_{\bar{S}} + \frac{\mu_{\bar{I}} \bar{\hbar}_{t-1}u_{t-1}^T}{N}$$

Compared to the SIR structure, the changes in this structure are the addition of 3 equations to represent the heterogeneity, split into two groups, including contact mixing between the two groups into the structure in $\mathcal{A}_t$, using $\hbar_{t-1} + \bar{\hbar}_{t-1}$ in the Bernoulli equations to calculate transmissions from both, multiplying the Bernoulli equation with $\hbar_{t-1}$ or $\bar{\hbar}_{t-1}$equations such that new infections are added only to their respective groups, and group specific rates of transitions. Without loss of generality, we can conclude that the ABENM structure can include heterogeneity.

Appendix IIc: Extension of the ABENM structure to model births and deaths

1. For diseases where persons develop immunity after infection, e.g., Susceptible(S)-Infectious(I)-Removed(R)-Deaths(D), or for chronic diseases, e.g., Susceptible(S)-Infectious(I)-Deaths(D):

For convenience of numerical testing between ABENM with ABNM, the main manuscript discussed a SIR structure for a closed population, i.e., no births, by presenting a model that tracked s_t, i_t, and r_t (the proportion of people who are Susceptible, Infected, and Removed/Deaths, respectively), over time t. For population-level modeling of reemerging disease outbreaks such as Measles or Ebola disease, where the assumption is that the epidemic would be mitigated within a short period of time, say a few months, the above structure would be sufficient. However, for population-level modeling of diseases that are chronic such as HIV, Hepatitis B, and Hepatitis C, where transmissions can occur over the duration of life of an infected person, it is necessary to consider a longer analytical horizon. Such a model should assume an open population, i.e., model births and deaths and populations aging over time.

The proposed ABENM structure is convenient for modeling an open population because the structure of the model keeps track of all contacts an infected person would have over their lifetime through the static adjacency matrix $\mathcal{A}_t$, while maintaining the activation and deactivation of contacts through the dynamic adjacency matrix V_t. Age would be modeled as a heterogeneous parameter (as in the previous section), by dividing the population into age-groups. Every time-unit, new susceptible persons would age into the first age-group in the compartmental model, over time transition to older age-groups, and age out through deaths. Infected persons in the network who are aging out can be kept track of as state Death(D) or deleted from the simulation. When a person becomes newly infected, the current age of one or more of their susceptible contacts could be outside the 'alive' susceptible population, e.g., a person who has aged-out could have been a partner in the past of currently alive persons and a person who has not yet entered the model (not yet aged-into) could be a contact in the future of a current alive person. We believe this framework is still computationally tractable. First, though age-groups outside the typical age-group range would need to be modeled, it would still be bounded. For example, if we take a maximum life-expectancy of 100 years, to keep track of all contacts of any alive person, in the most extreme case we would track ages -100 to +200. In cases such as HIV, this range would be narrow as the age difference between sexual partners are typically much lower. Second, as the ABENM only tracks infected persons and their immediate contacts, it is not computationally burdensome to keep track of all contacts during the lifetime of the person. Third, the computational complexity is still in the order of $O(N)$ (N=number of agents) as the lifetime contacts are determined only with respect to the infected node (and only once, at the time of infection) and tracked through the static adjacency matrix $\mathcal{A}_t$, while 'current' partnerships are modeled through the dynamic adjacency matrix V_t by setting its value to 1 or 0 to activate and deactivate the partnership.

In the above framework, determining the activation and deactivation times for each partnership, and the age of both partners at those time points would be key features to model, and would be done specific to the type of contacts, e.g., sexual partnerships in the US for HIV would be modeled using age-mixing between partners as they age. This is outside the scope of this manuscript, the application of the ABENM to HIV can be found be in [30](reference number from main paper). The authors use optimization methods for determining the activation and deactivation times of the partnerships, the age of the partners at the time of activation, and the current age of the partners. To generate the data needed for such a setup, they develop a Markov process model to simulate and extract longitudinal partnership changes over age, distributed by lifetime number of partners, from point estimates of population-level behavioral surveys. They further integrate a calibration process that uses national HIV surveillance data on changes in disease, behavior, and care parameters over time to fit the network and disease parameters representative of HIV in the US population.

We present below a general formulation for the population-level modeling that includes births and deaths, we first introduce additional parameters. Let,

B be the population renewal number, which could be interpreted as a birth rate multiplied by the population size, a constant number of births, or a rate or a number of persons aging into the susceptible population,

δ_S, δ_I, δ_R, be the mortality rate for Susceptible, Infected, and Removed, respectively and

$\mathcal{d}_t$ be a row vector of size Q_t taking binary values, 1 if a person is in D and 0 otherwise, and,

the rest of the parameters used in the SIR structure would remain the same, rewritten below for convenience,

$\mathcal{A}_t$ be a static adjacency matrix of size $Q_t \times Q_t$, where Q_t is the number of people to model at the individual-level, say persons in I and R and their immediate contacts.

V_t be a dynamic adjacency matrix of size $Q_t \times Q_t$

$\hbar_{\mathrm{t}}$ be a row vector of size Q_t taking binary values, 1 if a person is in I and 0 otherwise,

$\mathcal{m}_t$ be a row vector of size Q_t taking binary values, 1 if a person is in R and 0 otherwise,

$\mathcal{u}_{\mathrm{t}}$ be a unit array of size Q_t

μ_I be the rate of transitioning from I to R

μ_S be the rate of transitioning from S to R

c_{t} is a row vector, with $c_{\mathrm{t,j}}$ = the number of infected contacts of j if j is susceptible and alive, and = 0 otherwise, and given by $c_{\mathrm{t}} = (\mathcal{u} - \hbar_{t-1}) \circ (\mathcal{u} - \mathcal{m}_{t-1}) \circ (\mathcal{u} - \mathcal{d}_{t-1})\left((\mathcal{A}_{t-1} \circ V_{t-1})\, \hbar_{t-1}^T\right)^T$.

For this formulation of the model, which has an open population whose size could change over time, it would be more convenient to track the actual number of people in each state as S_t, I_t, R_t instead of tracking proportion of people in each state (s_t, i_t, and r_t) as introduced in the main manuscript for a closed population. Then, the initial equations introduced for the SIR model in the main manuscript would no more have the division by N (the population size). Additional changes include, an additional component denoting mortality subtracted in the equations of S_t, I_t, and R_t, specifically, $-S_{t-1}\delta_S$, $-\delta_I \hbar_{t-1} \mathcal{u}_{\mathrm{t}}^T$, and $-\delta_R \mathcal{m}_{t-1} \mathcal{u}_{\mathrm{t}}^T$, respectively, S_t would have an additional component (B) added to its equation, and there would be an equation tracking deaths, as follows.

$$S_t = \mathrm{B} + S_{t-1} - \sum_{j=1:Q_t} F^{-1}(1-(1-p)^{c_{t-1,j}}) - S_{t-1}\mu_S - S_{t-1}\delta_S$$

$$I_t = \hbar_{t-1}\mathcal{u}_{\mathrm{t}}^T + \sum_{j=1:Q_t} F^{-1}(1-(1-p)^{c_{t-1,j}}) - \mu_I \hbar_{t-1}\mathcal{u}_{\mathrm{t}}^T - \delta_I \hbar_{t-1}\mathcal{u}_{\mathrm{t}}^T$$

$$R_t = R_{t-1} + S_{t-1}\mu_S + \mu_I \hbar_{t-1}\mathcal{u}_{\mathrm{t}}^T - \delta_R \mathcal{m}_{t-1}\mathcal{u}_{\mathrm{t}}^T$$

$$D_t = D_{t-1} + S_{t-1}\delta_S + \delta_I \hbar_{t-1}\mathcal{u}_{\mathrm{t}}^T + \delta_R \mathcal{m}_{t-1}\mathcal{u}_{\mathrm{t}}^T$$

2. For diseases where persons can become re-infected, e.g., Susceptible(S)-Infectious(I)-Susceptible(S):

If the SIS is in the context of a low prevalence disease or early phases of an epidemic where the disease spreads through defined contact structures, they could still be tracked if they become re-susceptible.

For diseases such as seasonal flu (or COVID-19 if it is SIS), where the disease easily spreads through air droplets, or malaria and dengue, spread through high populations of the vector (mosquitoes), the resulting contact network would be equivalent to a random network, and thus, a compartmental model might be more suitable as it is equivalent to simulating a random network. For sexually transmitted diseases such as the human papilloma virus (HPV), chlamydia, gonorrhea, syphilis, or herpes, 50% to 80% of sexually active persons develop these diseases at least once in their lifetime. Therefore, network modeling could be used as the high prevalence does not create the same issues as discussed in the motivation of the ABENM or, as these diseases easily spread, a compartmental model could provide a good approximation. Other diseases that are of type SIS, where network structures are relevant, should be studied separately, and is outside the scope of our work."

Appendix III: Degree correlations between neighbor nodes on epidemic paths in networks

As discussed in the main manuscript, we hypothesize that conditional distributions derived for general scale-free networks cannot be used for determining the degree of the neighbors of the newly infected nodes in the ECNA network generation. Intuitively, the analytical expression for the conditional probability distribution derived on a general scale-free network (such as in (28)) would be representative of the distribution of degree of node neighbors of a randomly chosen set of nodes in the network. Empirically, the data for this can be generated by starting with one node, collecting their degree and the degree of each of their neighbors, and repeating this for all nodes. Therefore, if we consider nodes A and B in an undirected graph, the degree of A given degree of B and the vice-versa, i.e., the degree of B given the degree of A, are both incorporated into the estimation of the probability mass function. However, in the case of epidemics, the chance of A infecting B versus B infecting A would not be equal but vary as a function of the degree of A and B and the prevalence (proportion of population infected) at that time-point, thus creating directionality in flow (epidemic path) and making the chance of infection non-stationary as the prevalence changes over time, and should be thus considered in estimation of conditional distributions for ECNA. We present this more formally through Remarks 2 and 3 below

Remark 2*: The theoretical conditional distribution for degree correlations between neighbors, derived for general (non-contagion) networks, will generate biased estimates for the degree correlations between newly infected persons and their uninfected contacts in a contagion network*

Proof: We prove this by showing that the expected value of degree of the second-neighbors k of a node with degree d is different when considering all paths branching out of node d compared to when considering only a fraction of the paths branching out of node d. The former scenario represents the general estimation of degree correlations, as the combinations of all nodes and all their neighbors are used in the estimation. The latter scenario represents the proposed network generation algorithm where an infected node (A) may infect only a fraction of their first node neighbors, and so the degree of the second neighbors are dependent on the degree of the infected first node neighbors (we will refer to this combination of nodes as an epidemic path). Let's say one such pair of first and second neighbors of A are nodes B and C, respectively. Thus, the degree of a node C is determined as a function of degree of B only and none of the other neighbors of C. The mathematical representation is as follows.

Let,

D_i be a random variable denoting the degree of the ith neighbor of an infected node with degree d, e.g., suppose A is a contact of B, and B is a contact of C, then C is a second-neighbor of A,

$E_N[D_2|d]$ be the expected value of degree of second neighbors on 'any' randomly chosen path from a node of degree d in a given network N,

$E_{N,e_s}[D_2|d]$ be the expected value of degree of second neighbors on an 'epidemic path' e_s of a node of degree d in a network N; where $'s'$ in e_s denotes the assumptions of static contacts , i.e., all contacts of infected persons are equally exposed to the infection and, in Remark 5, denote epidemic paths as e_d to refer to the assumption of dynamic contacts,

$Pr\{D_2 = l|D_1 = k\}$ is the probability that the degree of D_2 (a second-neighbor) is l given degree of D_1 (a first neighbor) is k, and

$Pr\{ I_{D_1,e_s}\}$ be the probability that a first neighbor (D_1) becomes infected in a network with static contacts e_s

We can write

$$E_N[D_2|d] = \sum_{l=1:M} l \ Pr_N\{D_2 = l|d\}$$
$$= \sum_{l=1:M} l \sum_{k=1:M} Pr\{D_2 = \ l|D_1 = k\} \, Pr\{D_1 = k|d\} \ , \qquad (1)$$

where, $\sum_k Pr\{D_2 = \ l|D_1 = k\} \, Pr\{D_1 = k|d\}$ follows from the chain rule expansion of the conditional probability $Pr_N(D_2 = l|d)$ on a graph, i.e., for any node with degree d, all its second-neighbors D_2 pass through its first neighbors D_1, and M is the maximum node degree in the network.

Equivalently, for epidemic paths on contagion networks, we can write

$$E_{N,e_s}[D_2|d] = \sum_{l=1:M} l \, Pr_{N,e_s}\{D_2 = l|d\}$$
$$= \sum_{l=1:M} l \sum_{k=1:M} Pr\{D_2 = \ l|D_1 = k\} \mathrm{Pr}\{ I_{D_1,e_s}\} Pr\{D_1 = k|d\} \ , \qquad (2)$$

and $Pr\left\{ I_{D_1,e_s}\right\} = 1 - (1-p)^{c_j}; \ c_j = \sum_{q=1}^{k} \beta \ = k\beta,$

where,

$Pr\{ I_{D_1,e_s}\}$ is added to consider that a first neighbor D_1 will be on the epidemic path only if they become infected, and the equation for $Pr\left\{ I_{D_1,e_s}\right\}$ follows from using a Bernoulli process equation that evaluates the probability of disease transmission as 1 minus the probability of no transmission from any of its k contacts,

c_j is the number of infected contacts of j

β is the probability a contact is infected, and

p is the probability of transmission per infected-susceptible contact.

Note that if $p = 1, E_N[D_2|d] = E_{N,e_s}[D_2|d]$, and for $0 < p < 1$, $E_N[D_2|d] \neq E_{N,e_s}[D_2|d]$.

Therefore, while $Pr\{L = l|K = k\}$ is a good estimator for $Pr\{D_2 = \ l|D_1 = k\}$ for a randomly chosen path (as in non-contagion networks), it is not a good estimator for an epidemic path as $Pr\{L = l|K = k\} = Pr\{D_2 = \ l|D_1 = k\} Pr\left\{ I_{D_1,e_s}\right\}$, the term in the numerator of (2). This can be extended to any two D_i and D_{i+1}. Rewriting and substituting for $Pr\left\{ I_{D_1,e_s}\right\}$, on an epidemic path, for any 2 neighbors A and B with degree D_A and D_B, respectively, $Pr\{D_A = \ l|D_B = k\} = \frac{Pr\{L = l|K = k\}}{1-(1-p)^{k\beta}}, or$

$$Pr\{D_2 = \ l|D_1 = k\} > Pr\{L = l|K = k\} \ if \ \ p < 1; \ Pr\{D_2 = \ l|D_1 = k\} = Pr\{L = l|K = k\} \ if \ \ p = 1 \qquad (3)$$

thus suggesting that, for contagion networks, $Pr\{L = l|K = k\}$ is a biased estimator for $Pr\{D_A = \ l|D_B = k\}$.

This completes the proof.

Remark 3: *The distribution of degree of neighbors on epidemic paths are different for dynamic contagion networks compared to static contagion networks.*

Proof: While a static contagion network is one where all contacts of a node are equally exposed to the contagion at any time step, dynamic contagion networks are networks with dynamic contacts, e.g., in IDU networks individuals do not share needles with all contacts at each time step, and as such, not all contacts are equally exposed to the infection. Extending the proof in Remark 2, we show that $E_{N,e_s}[D_2|d] \neq E_{N,e_d}[D_2|d]$, where

$E_{N,e_d}[D_2|d]$ is the expected value of degree of second neighbors of a node with degree d on epidemic paths with dynamic contacts (e_d) on a network N, and written as

$$E_{N,e_d}[D_2|d] = \sum_{l=1:M} l \sum_{k=1:M} Pr\{D_2 = l|D_1 = k\} Pr\{I_{D_1,e_d}\} Pr\{D_1 = k|d\} \quad (4)$$

$$Pr\{I_{D_1,e_d}\} = 1 - (1-p)^{c_j} \,;\, c_j = \sum_{q=1}^{k} \beta \frac{1}{d_q}$$

Note that $Pr\{I_{D_1,e_d}\}$ is similar to $Pr\{ I_{D_1,e_s}\}$ used in Remark 2, except for the additional component $\frac{1}{d_q}$ where d_q is the degree of q. Here, without loss of generality, we are assuming that each of q's contacts have an equal chance of being active and thus $\frac{1}{d_q}$ is a proxy for the probability that D_1 is an active contact of q, but the concept can be applied to any other assumptions for contact activation by modifying this equation. Thus, if $p = 1, E_N[D_2|d] = E_{N,e_s}[D_2|d] = E_{N,e_d}[D_2|d]$, and for $0 < p < 1$, $E_N[D_2|d] \neq E_{N,e_s}[D_2|d] \neq E_{N,e_d}[D_2|d]$.

Thus, in a static contagion network, the probability that a susceptible person becomes infected is solely reliant on the degree (k) of the susceptible node. However, in a dynamic contagion network, the probability of infection is also dependent on the degree of each of those k nodes. And further, for both networks, the probability a node becomes infected is directly proportional to its degree k and, additionally, for dynamic networks, indirectly proportional to its neighbors' degree.

Therefore, as in Remark 2, $Pr\{D_2 = l|D_1 = k\} > Pr\{L = l|K = k\}$ if $p < 1$; $Pr\{D_2 = l|D_1 = k\} = Pr\{L = l|K = k\}$ if $p = 1$

This completes the proof.

Appendix IV: Scale-free network distributions

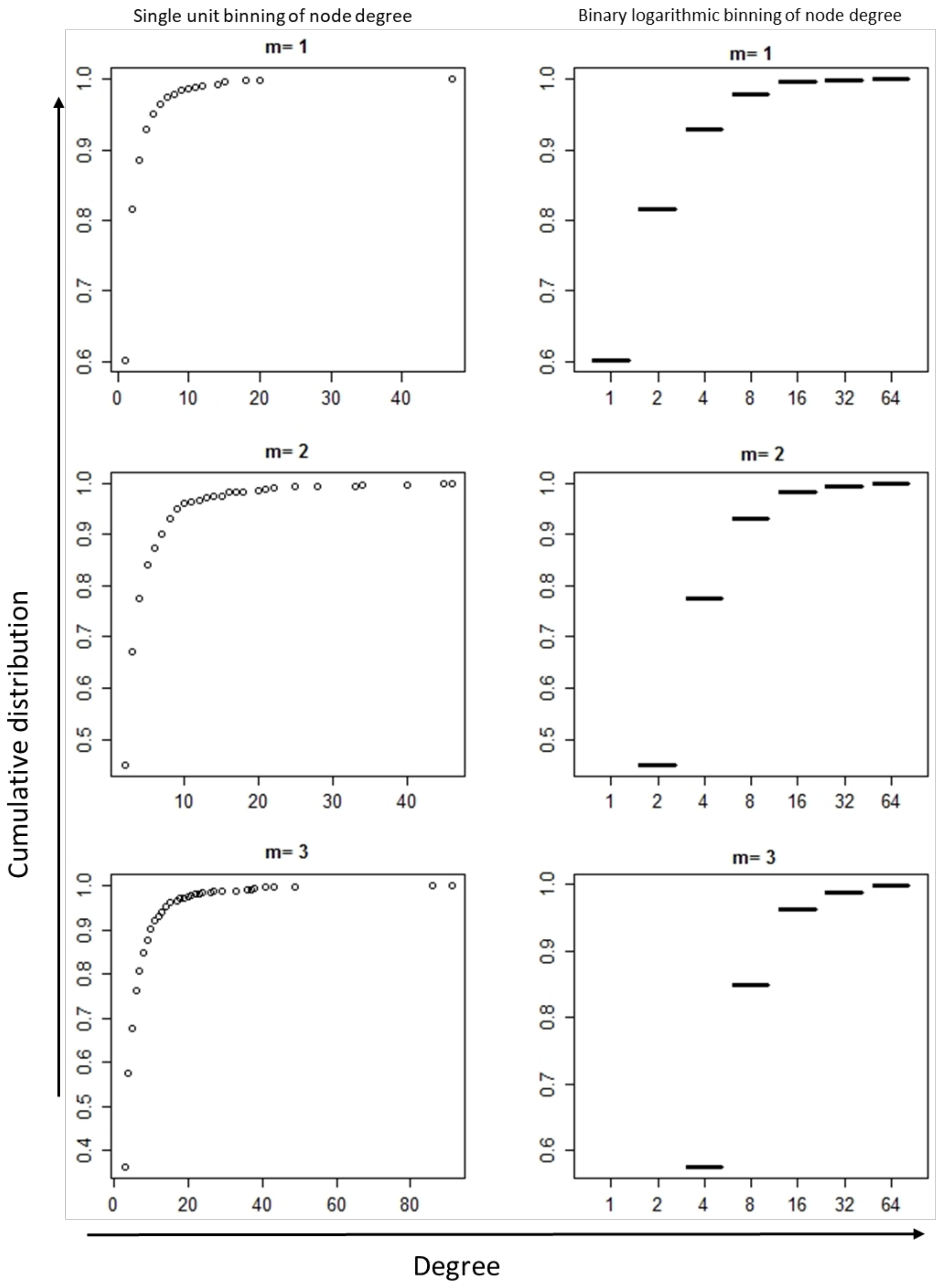

Figure IVa: Degree distribution of scale-free networks of size 1000 nodes under different values of minimum degree m. Scale-free networks follow a power-law distribution. The characteristic feature of power-law distributions is that a very small number of nodes will have a large degree and most nodes have a small degree

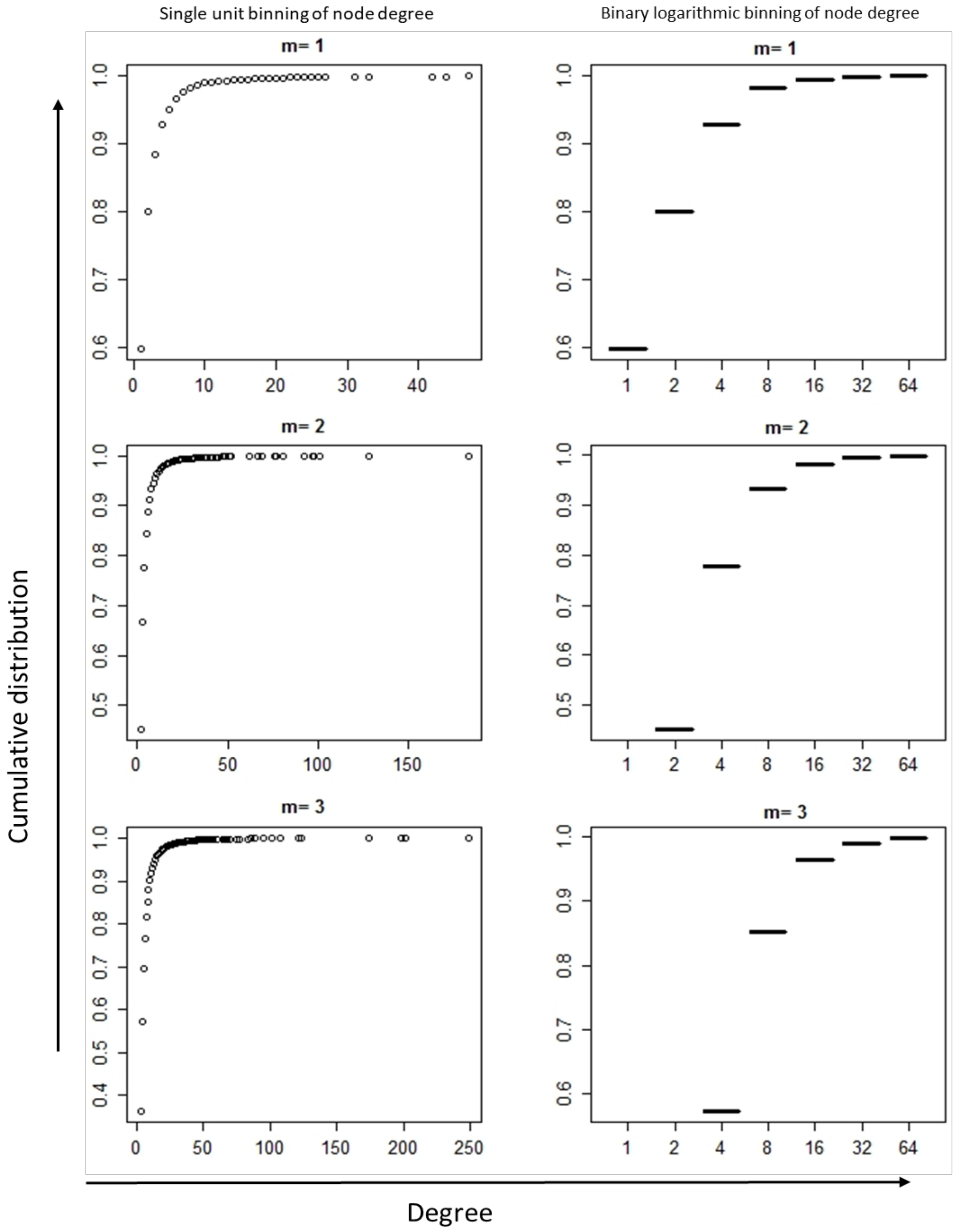

Figure IVb: Degree distribution of scale-free networks of size 10000 nodes under different values of minimum degree m. Scale-free networks follow a power-law distribution. The characteristic feature of power-law distributions is that a very small number of nodes will have a large degree and most nodes have a small degree

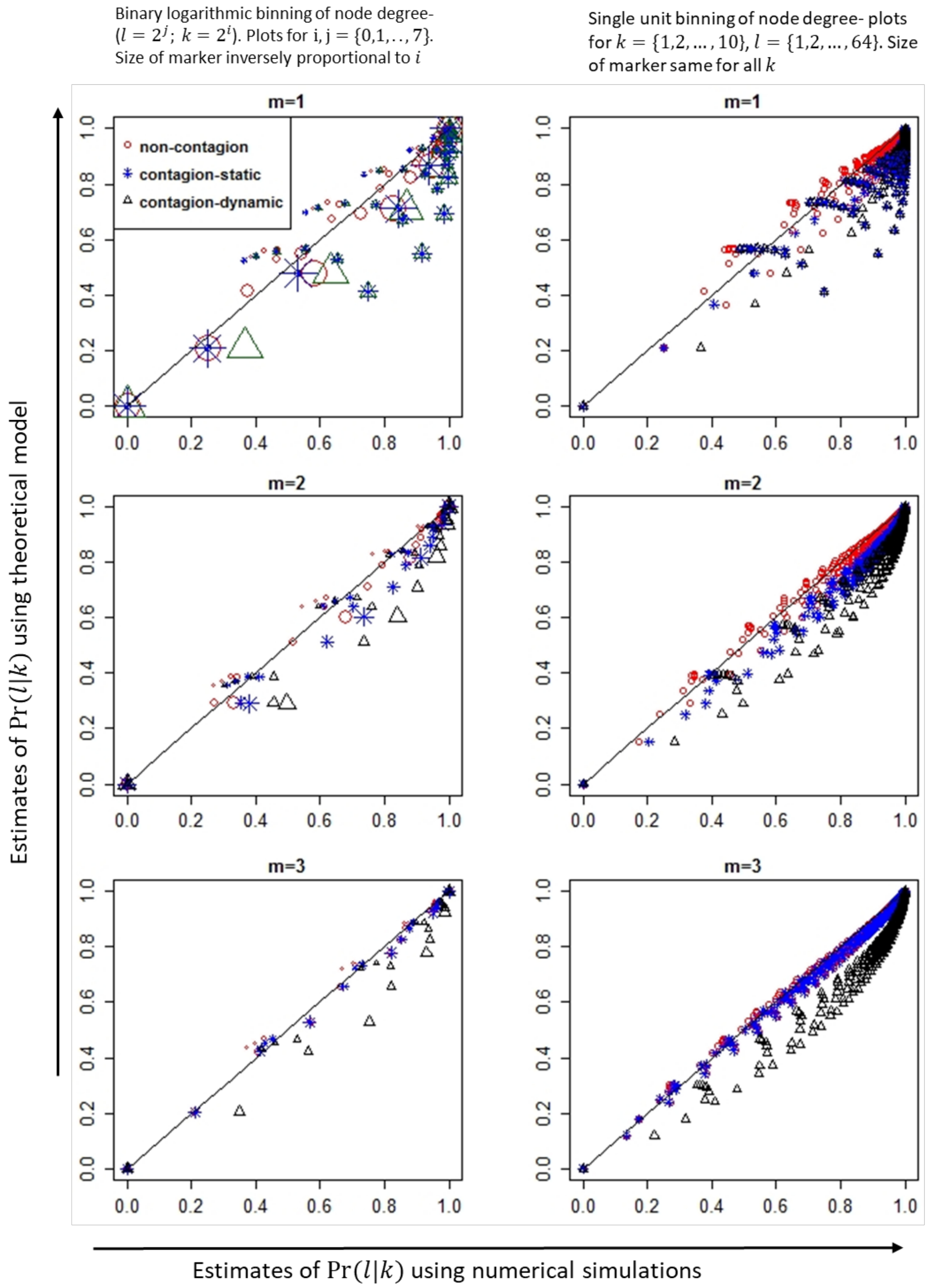

Figure IVc: Comparing numerically estimated degree correlations on non-contagion and contagion networks with theoretically known distributions of degree correlations. $Pr(l|k)$ is the probability that given a node of degree k, the degree of its neighbor is l. Theoretical estimates are from model in Equation (10), and numerical estimates are from ABNM simulations. Results are from networks of size 10,000. The main manuscript presents results for networks of size 1000.

Appendix V: Neural network predictions for degree correlation on test networks

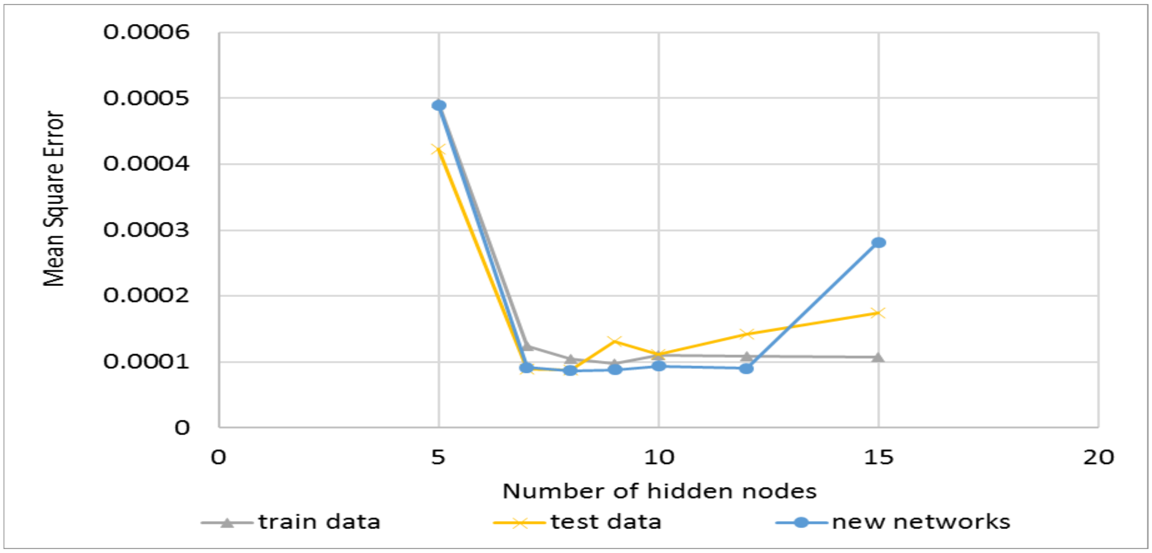

Figure Va: Tuning of the neural network hyperparameter, number of hidden nodes- Networks of combinations of sizes (s) 1000, 5000, and 10,000 and minimum degree (m) of 1 to 5 were generated. Networks were split into test and train networks, all networks except s1000m1, s5000m2, and s10000m4, were set as train networks. Train networks were further split into 60% and 40% train and test data, respectively, through random selection. Only train data of train networks were used in NN prediction. The graph shows the mean square errors (MSE) of neural network (NN) predictions as a function of the hyper-parameter. As expected, while the MSE decreases with increase in hidden nodes in the train data, in test data and test networks it decreases and then starts to increase after 8 hidden nodes. Therefore, we set the NN hyperparameter value at 8 hidden nodes

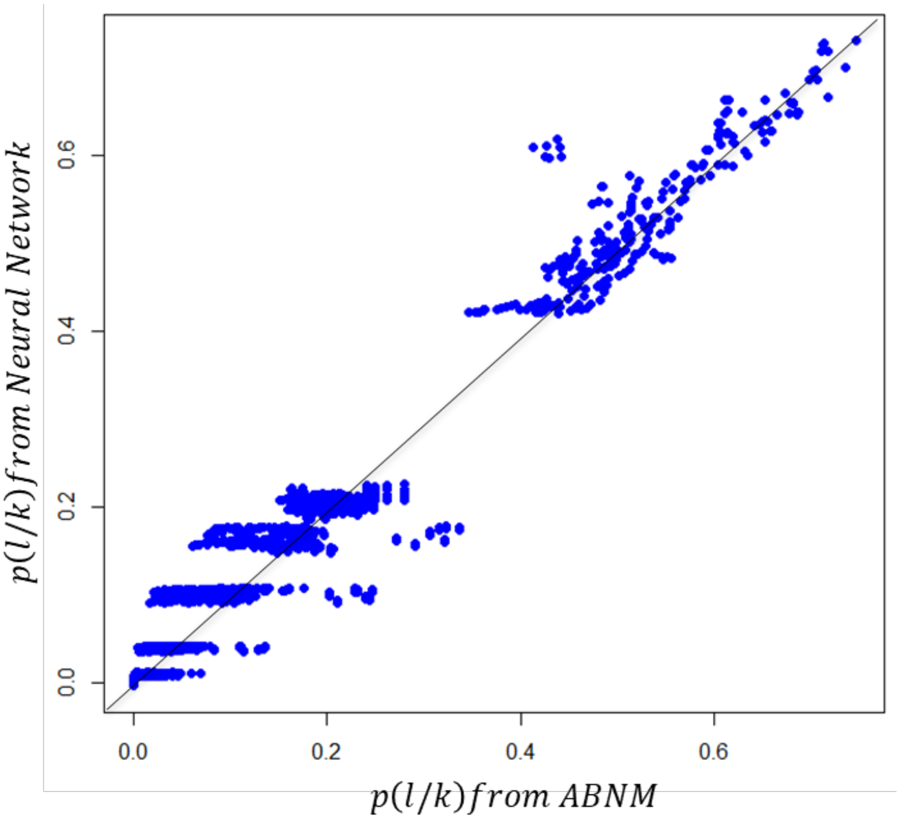

Figure Vb: A neural network model was trained to predict degree correlations defined by the conditional probability, $p(l|k)$ (given that a node has degree k, the probability its neighbors degree is l), using various scale-free networks defined by different lambda values. Using an agent-based network model (ABNM) actual conditional degree distribution were numerically recorded at specific proportion infected. By comparing the predicted response of the NN versus actual data in the ABNM, we can visually inspect that the NN is predicting within a certain accuracy. To make sure that the neural network is not over trained, this graph was generated using networks from test data set, i.e., networks not used in training, including values for the proportion infected that were not used in the training set of the NN.

Appendix VI: Sensitivity analysis - Results for epidemic predictions under varying values of minimum degree, transmission probability, and initial infection on networks of size 10,000 (Susceptible-Infected (SI) epidemic)

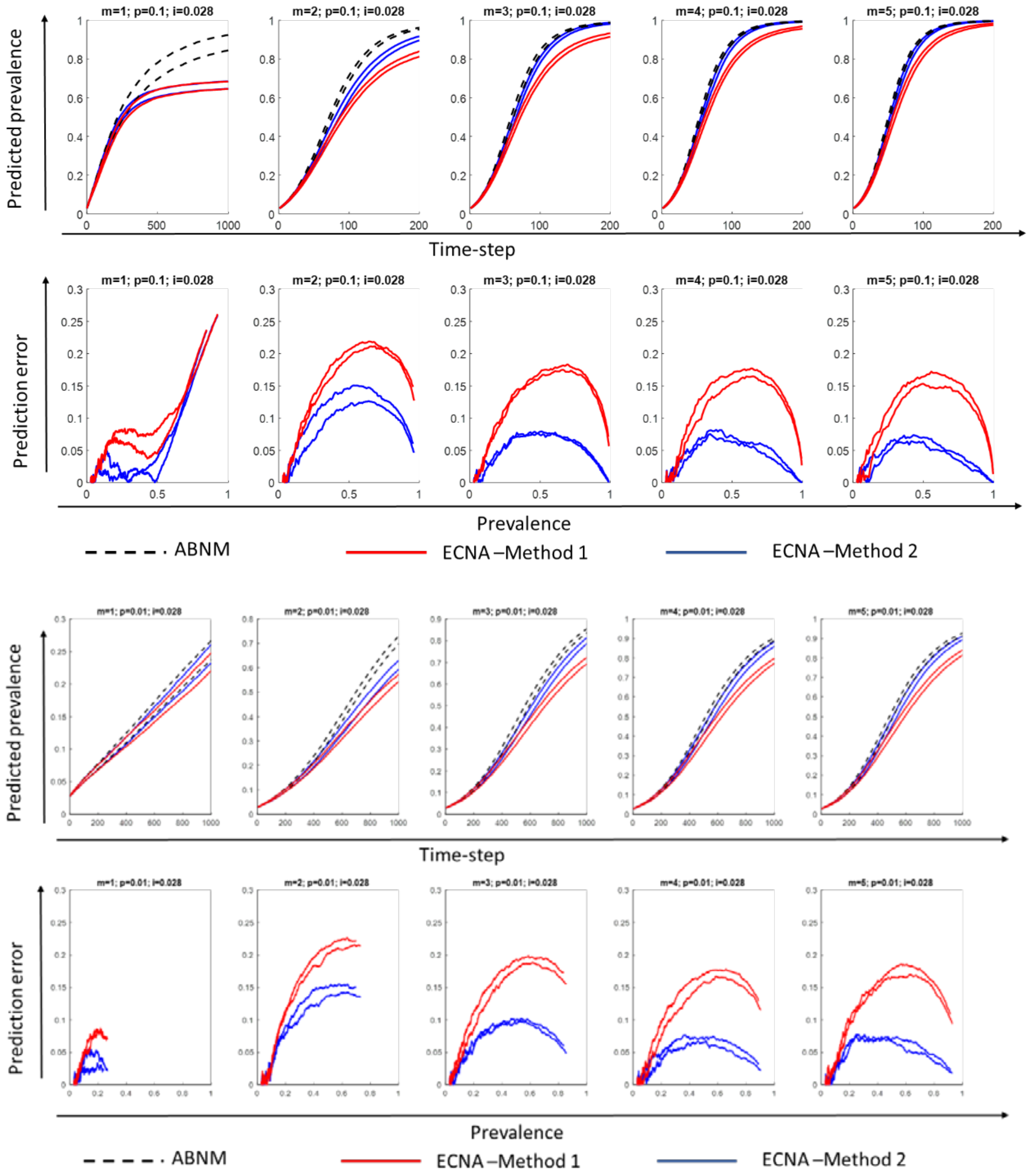

Figure VIa: Disease prevalence (proportion of population infected) predictions and prediction errors in ABENM (ECNA Methods 1 and 2) compared to ABNM for networks with minimum degree m = 1 to 5, **transmission probability per exposure $\boldsymbol{p = 0.1}$ and 0.01**, **initial proportion infected i = 0.028**, and **network size N = 10000**; Plots show the 5th and 95th percentile values of 100 runs**.** ABNM: Agent-based network model; ABENM: Agent-based evolving network model; ECNA- Evolving contact network algorithm; Method 1: Using theoretically known degree correlations between neighbors (eq. [10]). Method 2: Using neural network predictions for modified degree correlations between neighbors on epidemic paths in dynamic contagion networks.

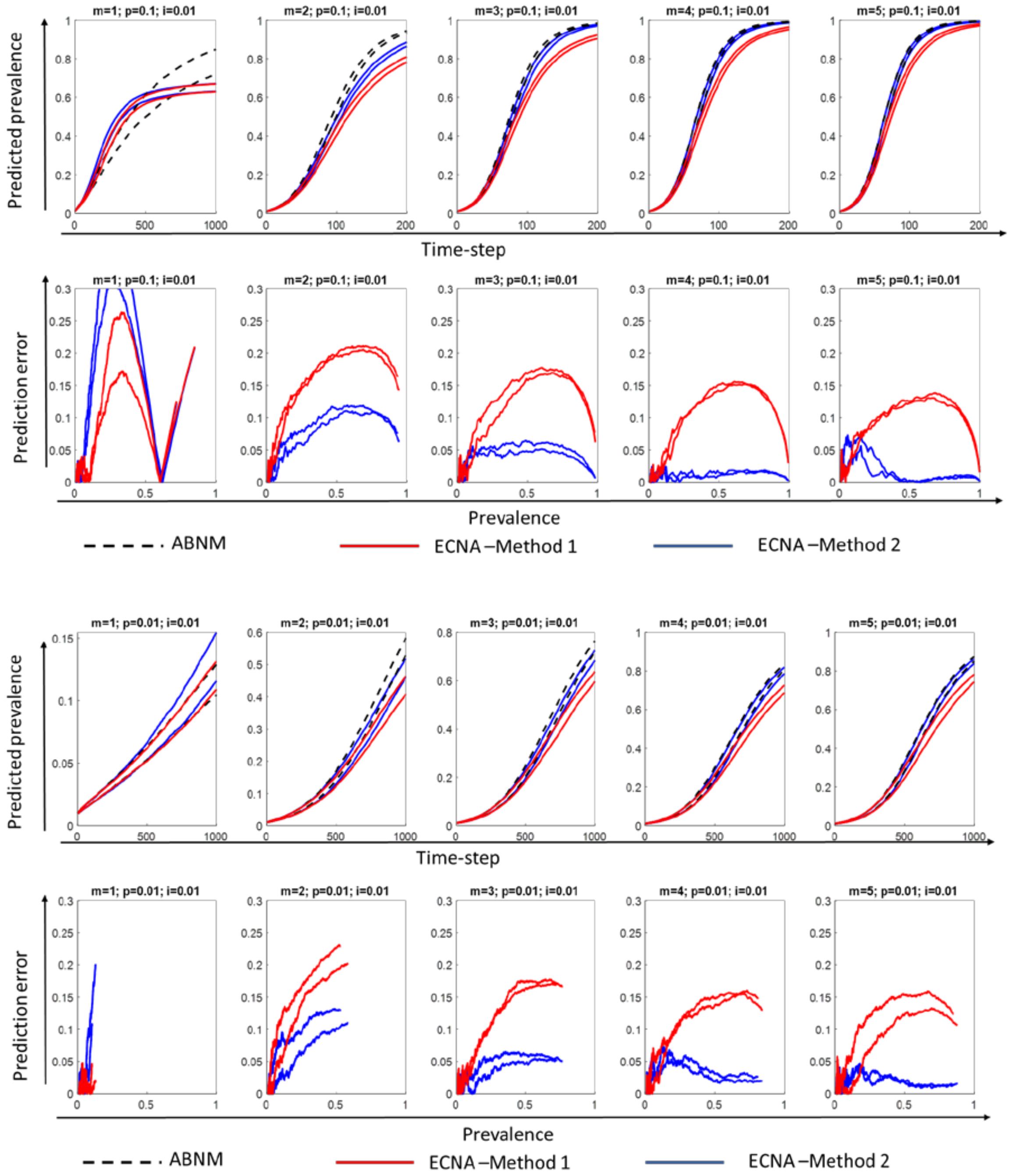

Figure VIb: Disease prevalence (proportion of population infected) predictions and prediction errors in ABENM (ECNA Methods 1 and 2) compared to ABNM for networks with **minimum degree m = 1 to 5, transmission probability per exposure $p = 0.1$ and 0.01**, **initial proportion infected i = 0.01**, and **network size N = 10000**; Plots show the 5th and 95th percentile values of 100 runs**.** ABNM: Agent-based network model; ABENM: Agent-based evolving network model; ECNA- Evolving contact network algorithm; Method 1: Using theoretically known degree correlations between neighbors (eq. [10]). Method 2: Using neural network predictions for modified degree correlations between neighbors on epidemic paths in dynamic contagion networks.

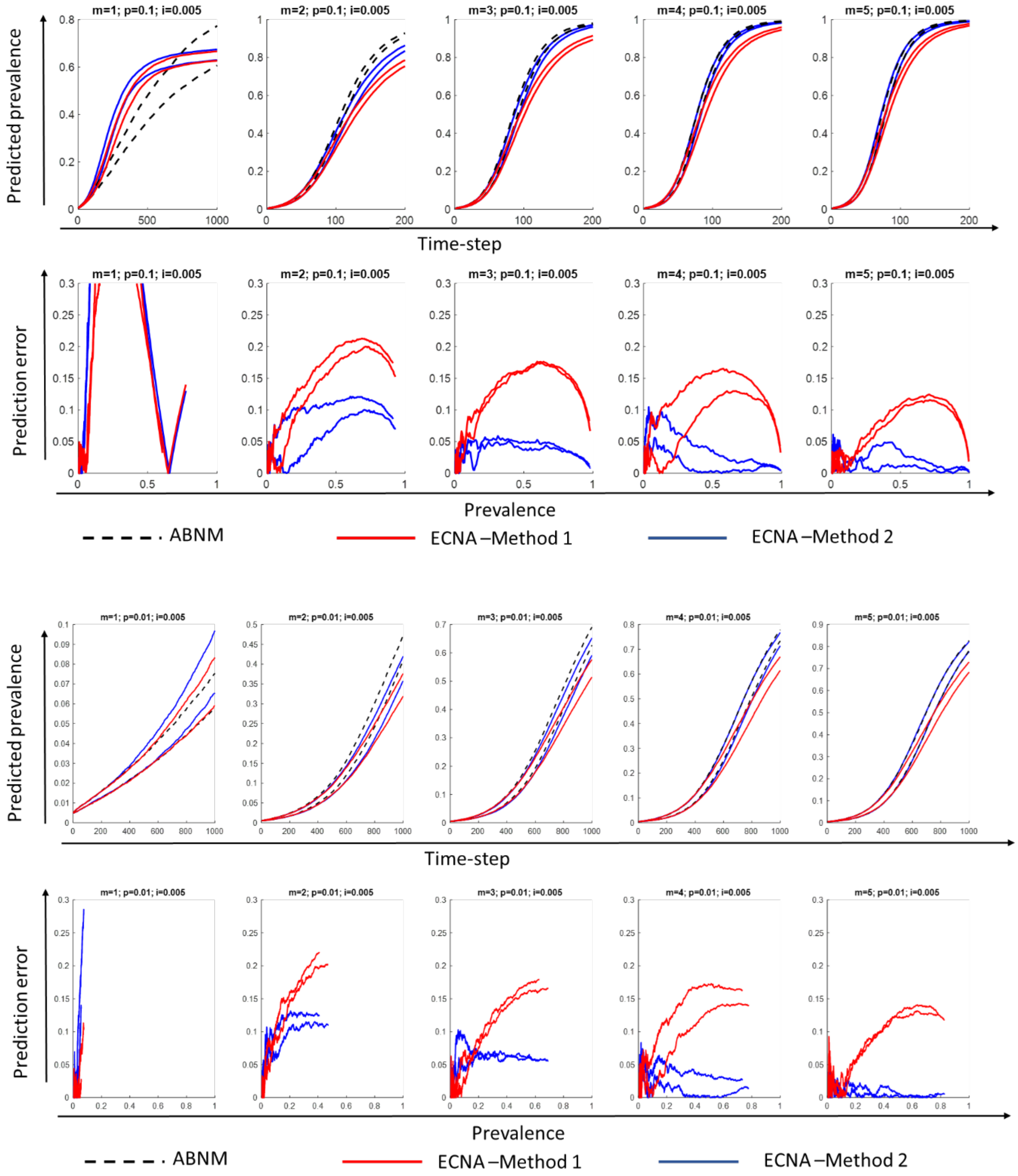

Figure VIc: Disease prevalence (proportion of population infected) predictions and prediction errors in ABNEM (ECNA Methods 1 and 2) compared to ABNM for networks with minimum degree m = 1 to 5, **transmission probability per exposure $\boldsymbol{p = 0.1}$ and 0.01**, **initial proportion infected i = 0.005**, and **network size N = 10000**; Plots show the 5th and 95th percentile values of 100 runs**.** ABNM: Agent-based network model; ABENM: Agent-based evolving network model; ECNA- Evolving contact network algorithm; Method 1: Using theoretically known degree correlations between neighbors (eq. [10]). Method 2: Using neural network predictions for modified degree correlations between neighbors on epidemic paths in dynamic contagion networks.

Appendix VII: Sensitivity analysis - Results for epidemic predictions under varying values of minimum degree, transmission probability, and initial infection on networks of size 1000 (Susceptible-Infected (SI) epidemic)

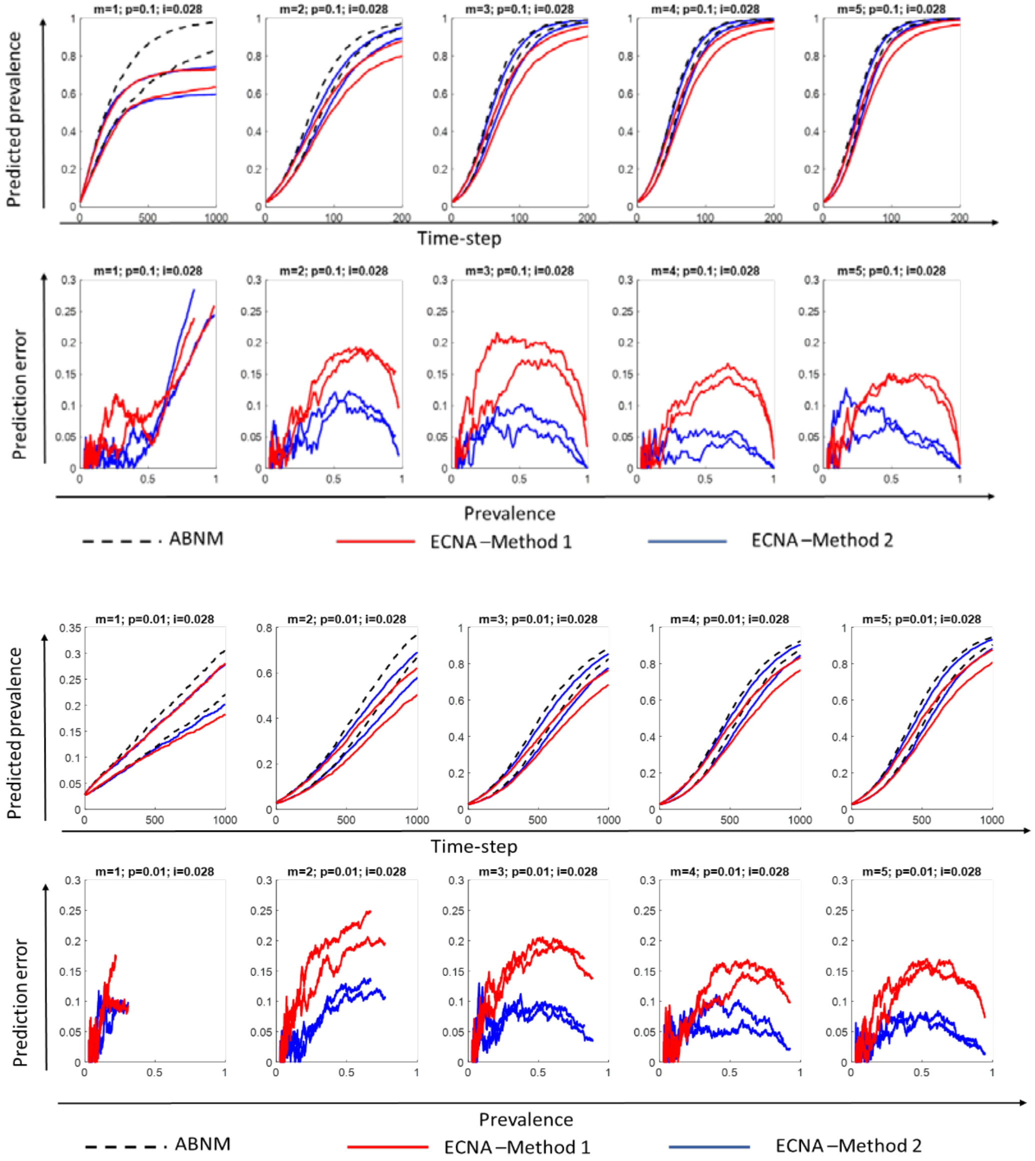

Figure VIIa: Disease prevalence (proportion of population infected) predictions and prediction errors in ABENM (ECNA Methods 1 and 2) compared to ABNM for networks with **minimum degree m = 1 to 5, transmission probability per exposure $p = 0.1$ and 0.01, initial proportion infected i = 0.028, and network size N = 1000**; A Plots show the 5th and 95th percentile values of 100 runs**.** ABNM: Agent-based network model; ABENM: Agent-based evolving network model; ECNA- Evolving contact network algorithm; Method 1: Using theoretically known degree correlations between neighbors (eq. [10]). Method 2: Using neural network predictions for modified degree correlations between neighbors on epidemic paths in dynamic contagion networks.

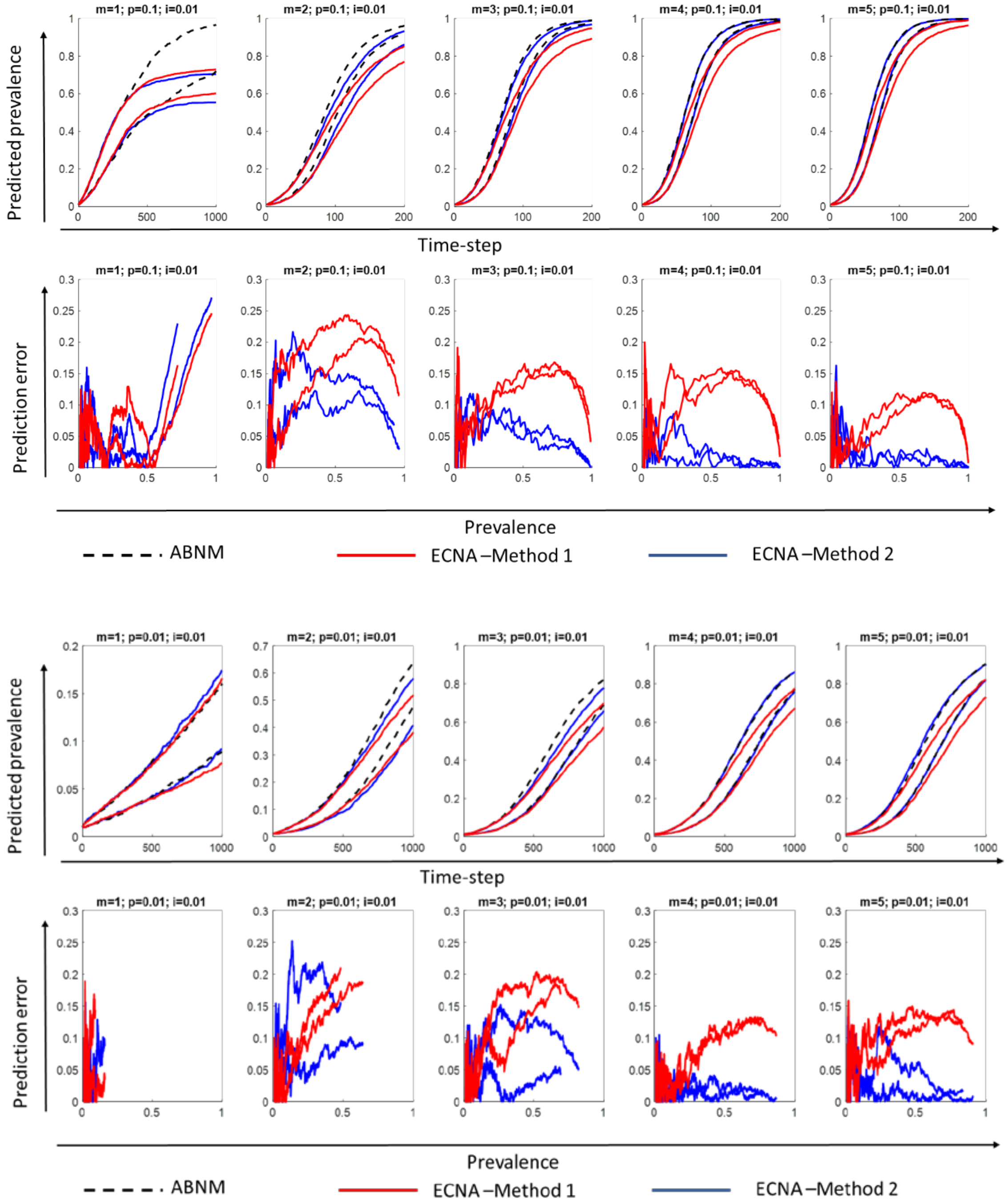

Figure VIIb: Disease prevalence (proportion of population infected) predictions and prediction errors in ABENM(ECNA Methods 1 and 2) compared to ABNM for networks with **minimum degree m = 1 to 5, transmission probability per exposure $p = 0.1$ and 0.01, initial proportion infected i = 0.01, and network size N = 1000**; Plots show the 5th and 95th percentile values of 100 runs**.** ABNM: Agent-based network model; ABENM: Agent-based evolving network model; ECNA- Evolving contact network algorithm; Method 1: Using theoretically known degree correlations between neighbors (eq. [10]). Method 2: Using neural network predictions for modified degree correlations between neighbors on epidemic paths in dynamic contagion networks.

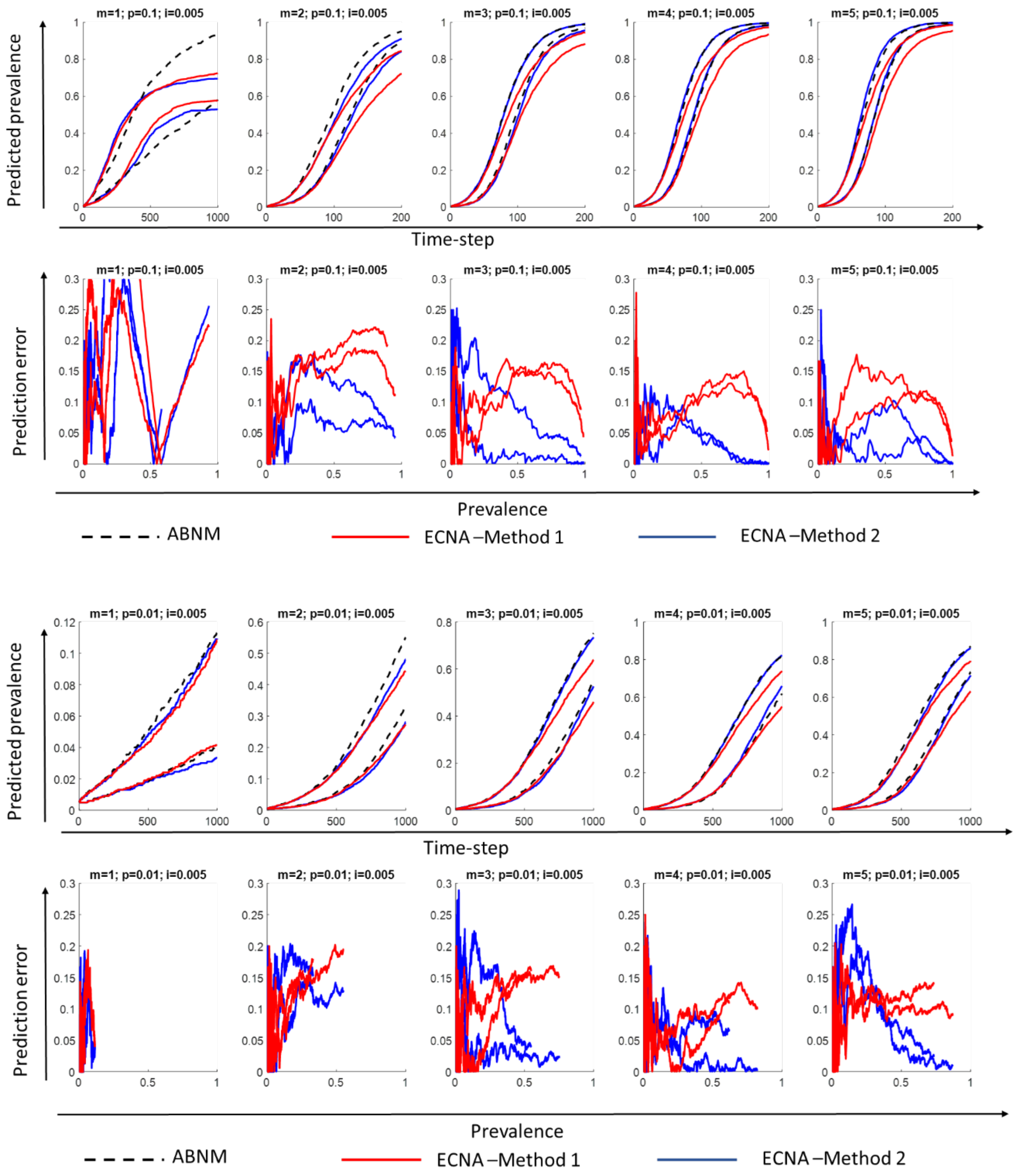

Figure VIIc: Disease prevalence (proportion of population infected) predictions and prediction errors in ABENM (ECNA Methods 1 and 2) compared to ABNM for networks **with minimum degree m = 1 to 5, transmission probability per exposure $p = 0.1$ and 0.01, initial proportion infected i = 0.005, and network size N = 1000;** Plots show the 5th and 95th percentile values of 100 runs**.** ABNM: Agent-based network model; ABENM: Agent-based evolving network model; ECNA- Evolving contact network algorithm; Method 1: Using theoretically known degree correlations between neighbors (eq. [10]). Method 2: Using neural network predictions for modified degree correlations between neighbors on epidemic paths in dynamic contagion networks.

Appendix VIII: Sensitivity analysis – Random values of transmission probability (Susceptible-Infected (SI) epidemic)

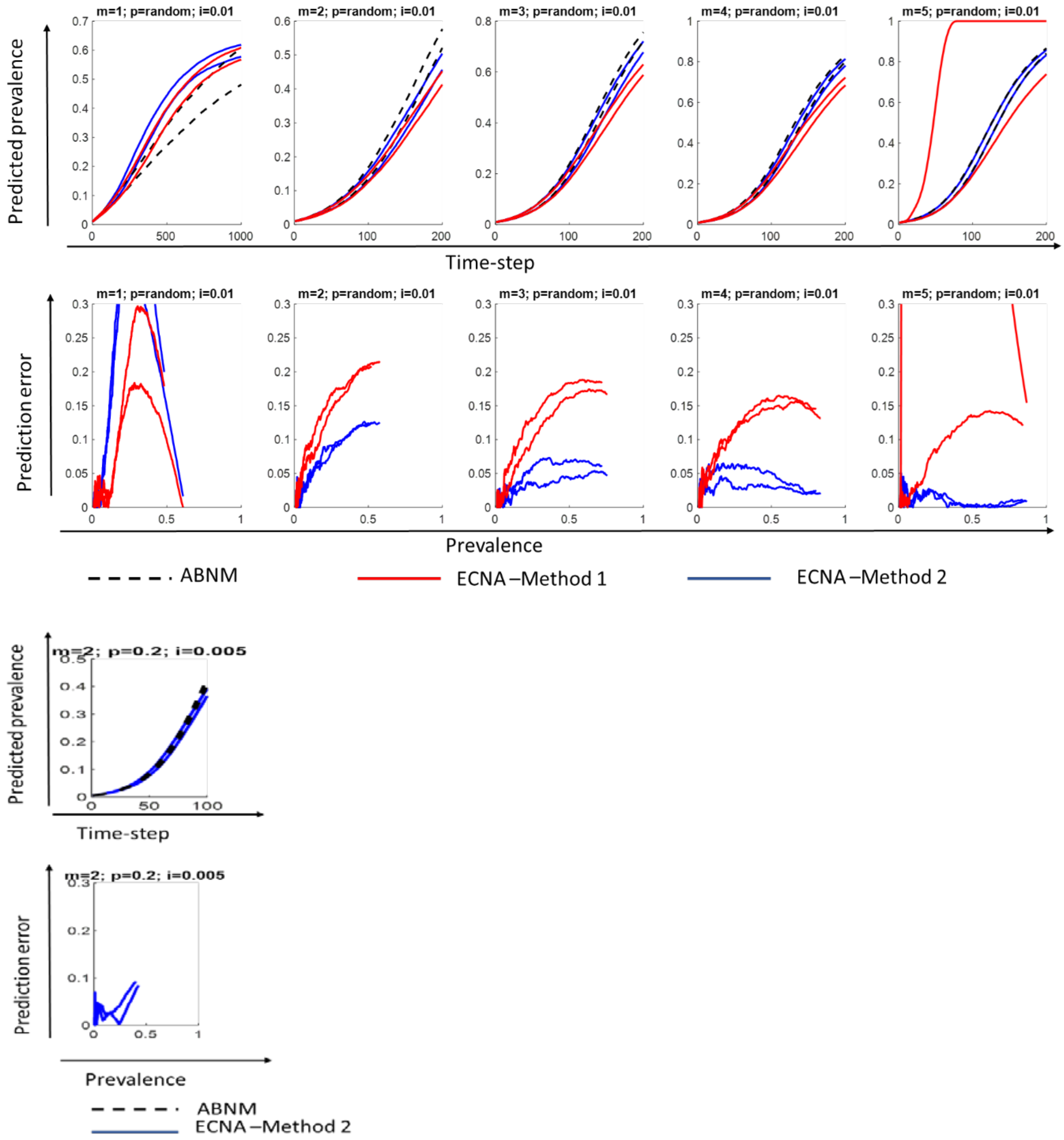

Figure VIIa: Disease prevalence (proportion of population infected) predictions and prediction errors in ABENM (ECNA Method 2) compared to ABNM for networks **with minimum degree m = 1 to 5, transmission probability per exposure $p = U[0, 0.1]$, initial proportion infected i = 0.01, and network size N = 10000 (top);** and **m = 2, $p = U[0, 0.2]$, i = 0.005, and N = 50000 (bottom);** Plots show the 5th and 95th percentile values of 100 runs**.** ABNM: Agent-based network model; ABENM: Agent-based evolving network model; ECNA- Evolving contact network algorithm; Method 1: Using theoretically known degree correlations between neighbors (eq. [10]). Method 2: Using neural network predictions for modified degree correlations between neighbors on epidemic paths in dynamic contagion networks; $U[a, b]$: continuous uniform distribution between values a and b.

Appendix IX: Sensitivity analysis – Susceptible-Infected-Recovered (SIR) epidemic

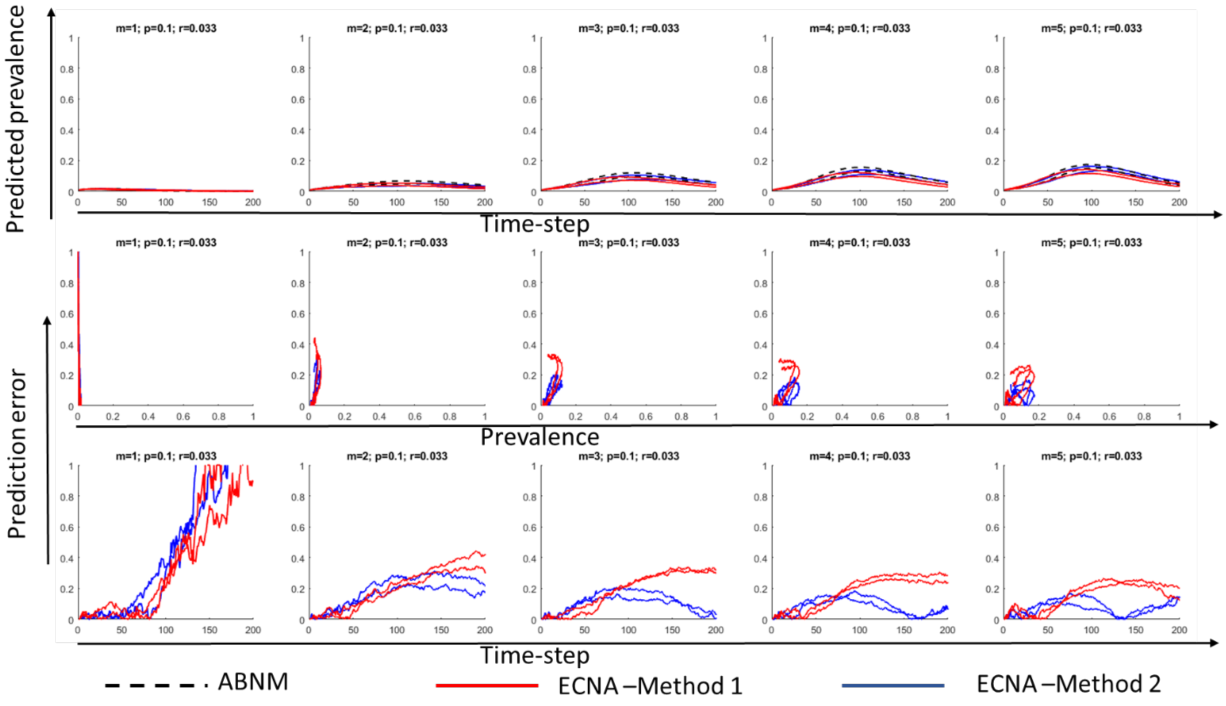

Figure IXa: transmission probability per exposure $p = 0.1$, and recovery rate r = 0.033

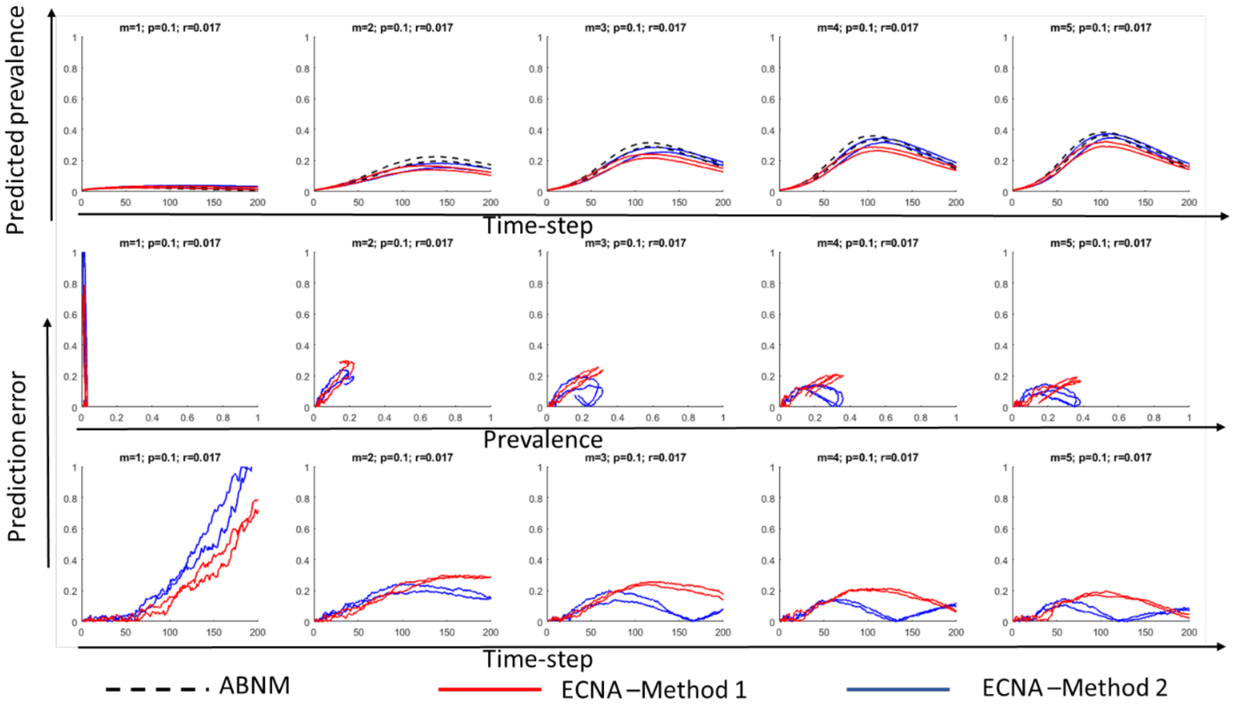

Figure IXb: transmission probability per exposure $p = 0.1$, and recovery rate r = 0.017

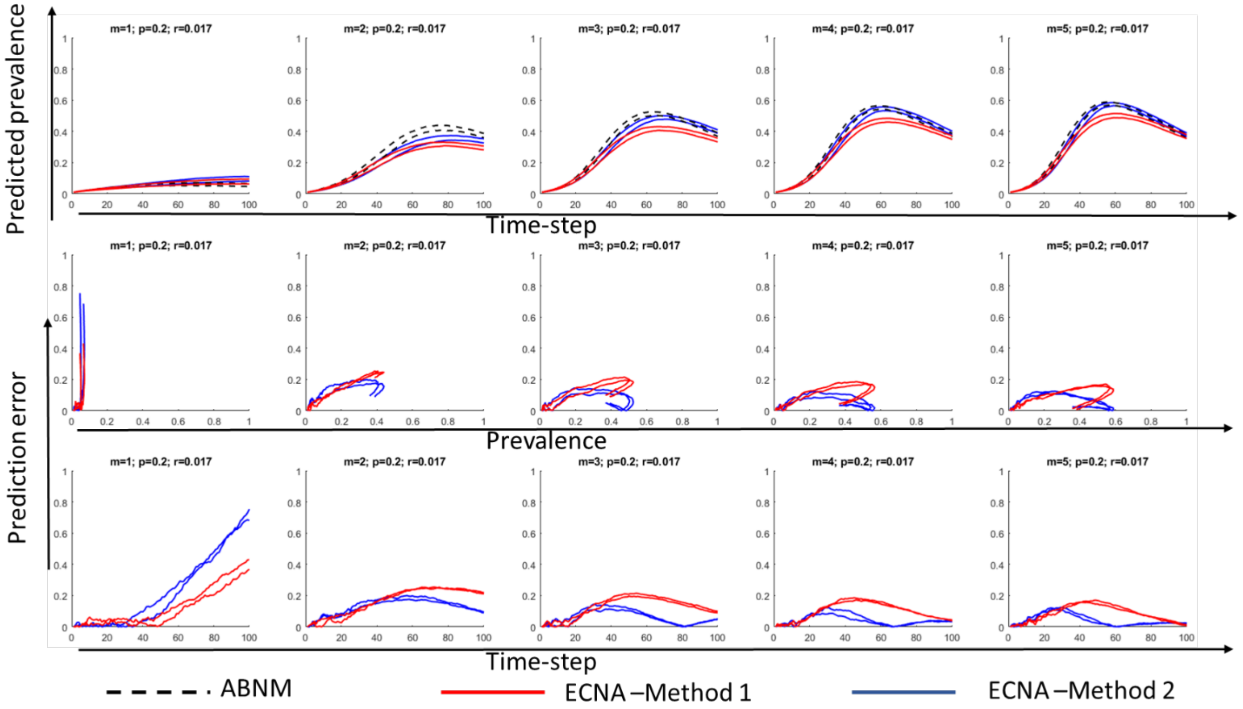

Figure IXc: transmission probability per exposure $p = 0.2$, and recovery rate r = 0.017

Figure IX**: SIR epidemic profile**: Disease prevalence (proportion of population infected) predictions and prediction errors in ABENM (ECNA Methods 1 and 2) compared to ABNM for networks with minimum degree m = 1 to 5, initial proportion infected i = 0.01, and network size N = 10000 for **IXa: p= $\mathbf{0.1}$, r = 0.033; IXb: p= $\mathbf{0.1}$, r = 0.017; IXc: p= $\mathbf{0.2}$, r = 0.017.** Plots show the 5th and 95th percentile values of 100 runs; p= transmission probability per exposure; r= recovery rate; ABNM: Agent-based network model; ABENM: Agent-based evolving network model; ECNA- Evolving contact network algorithm; Method 1: Using theoretically known degree correlations between neighbors (eq. [10]). Method 2: Using neural network predictions for modified degree correlations between neighbors on epidemic paths in dynamic contagion networks; **As prevalence does not necessarily increase by time for SIR, prediction errors are presented both as a function of prevalence and time.**

Appendix X: Sensitivity analysis – Susceptible-Infected-Susceptible (SIS) epidemic

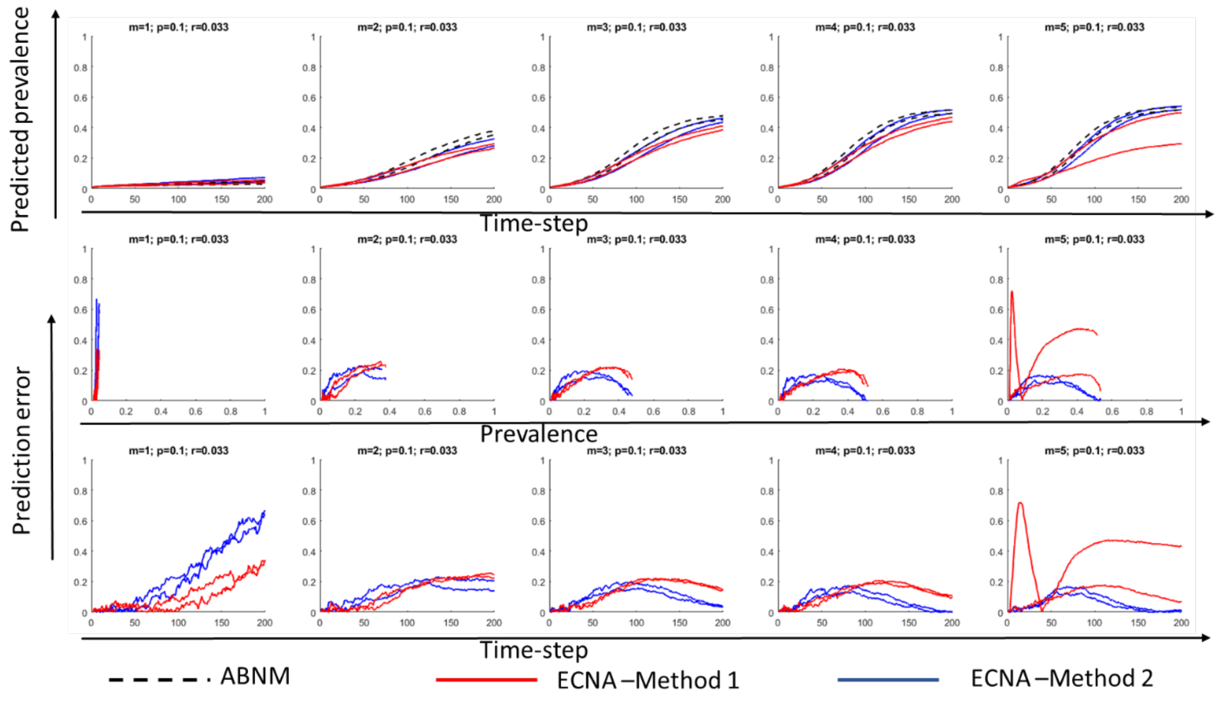

Figure Xa: transmission probability per exposure $p = 0.1$, and recovery rate r = 0.033

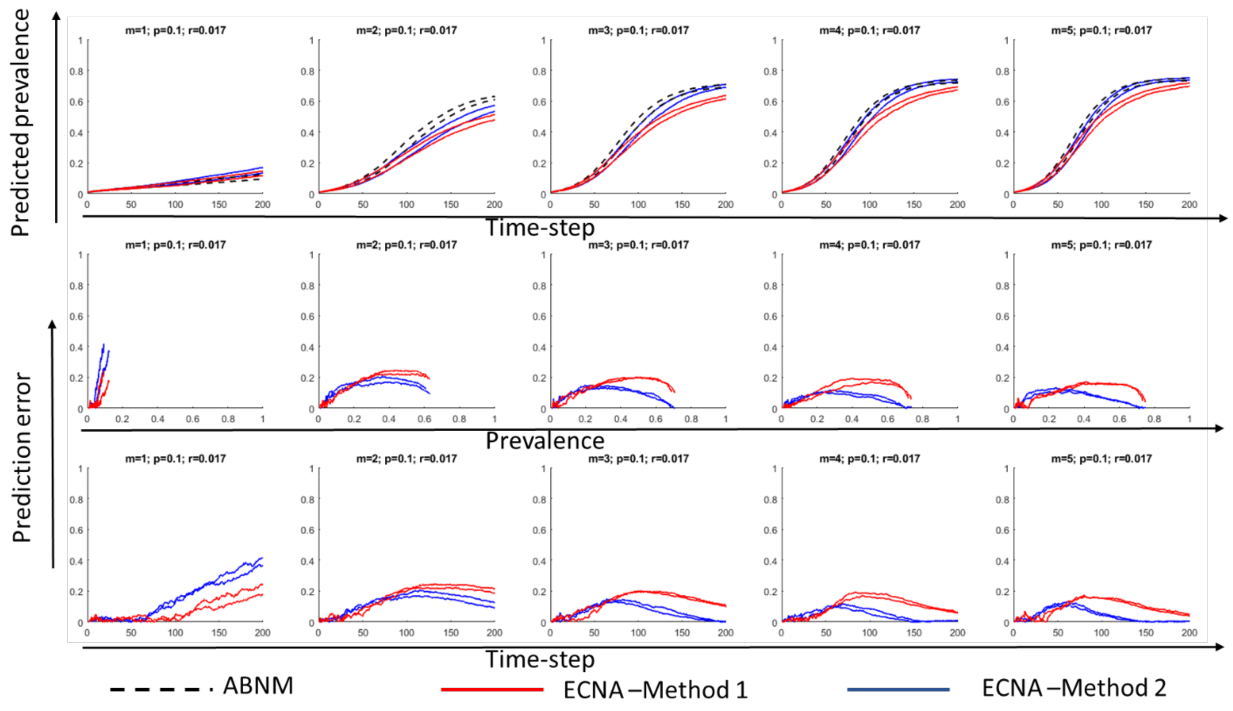

Figure Xb: transmission probability per exposure $p = 0.1$, and recovery rate r = 0.017

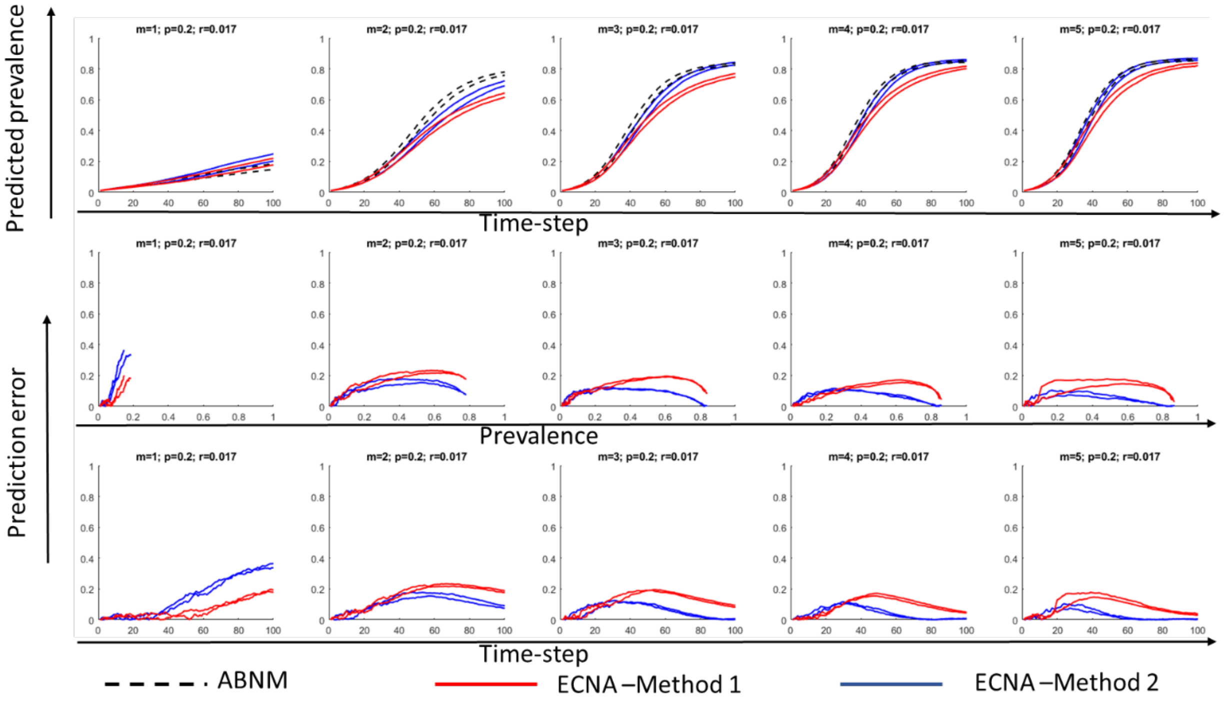

Figure Xc: transmission probability per exposure $p = 0.2$, and recovery rate r = 0.017

Figure X**: SIS epidemic profile**: Disease prevalence (proportion of population infected) predictions and prediction errors in ABENM (ECNA Methods 1 and 2) compared to ABNM for networks with minimum degree m = 1 to 5, initial proportion infected i = 0.01, and network size N = 10000 for **Xa: p= 0.1, r = 0.033; Xb: p= 0.1, r = 0.017; Xc: p= 0.2, r = 0.017.** Plots show the 5th and 95th percentile values of 100 runs; p= transmission probability per exposure; r= recovery rate; ABNM: Agent-based network model; ABENM: Agent-based evolving network model; ECNA- Evolving contact network algorithm; Method 1: Using theoretically known degree correlations between neighbors (eq. [10]). Method 2: Using neural network predictions for modified degree correlations between neighbors on epidemic paths in dynamic contagion networks; **As prevalence does not necessarily increase by time for SIS, prediction errors are presented both as a function of prevalence and time.**
